# Impact of Anthropogenic Emission Estimates on Air Quality and Human Health Effects

**DOI:** 10.1029/2024GH001223

**Published:** 2025-10-21

**Authors:** Halima Salah, Ying Xiong, Debatosh Partha, Noribeth Mariscal, Like Wang, Simone Tilmes, Wenfu Tang, Yaoxian Huang

**Affiliations:** ^1^ Department of Civil and Environmental Engineering Wayne State University Detroit MI USA; ^2^ Now at College of Environment and Climate, Institute for Environmental and Climate Research Guangdong‐Hongkong‐Macau Joint Laboratory of Collaborative Innovation for Environmental Quality, Jinan University Guangzhou China; ^3^ Now at Department of Earth, Environmental and Planetary Sciences Northwestern University Evanston IL USA; ^4^ Now at Max‐Planck‐Institute for Meteorology Hamburg Germany; ^5^ NSF National Center for Atmospheric Research Boulder CO USA

## Abstract

Global bottom‐up anthropogenic emission inventories show substantial spatial and temporal differences of short‐lived pollutant emissions, which results in uncertainties in terms of air quality and human health impacts. In this study, we compare the emissions of trace gases and aerosols for the year 2015 from three different global emission inventories, the Community Emissions Data System (CEDS), the Copernicus Atmosphere Monitoring Service Global Anthropogenic Emissions (CAMS‐GLOB‐ANT), and Evaluating the Climate and Air Quality Impacts of Short‐Lived Pollutants version 6b (ECLIPSEv6b). We then employ the Community Atmosphere Model with chemistry version 6.0 within the Community Earth System Model version 2.2.0 to quantify the atmospheric chemistry and air quality impacts from the above three anthropogenic emission inventories, with a focus on PM_2.5_ (particulate matter with aerodynamic diameters equal or less than 2.5 μm) and ozone (O_3_). Our results indicate that differences between emission inventories are largest for black carbon, organic carbon, ammonia and sulfur dioxide, in terms of global annual total emissions. These differences in emissions across CEDS, CAMS, and ECLIPSEv6b lead to substantial variations in global annual totals and spatial distribution patterns. This study shows that the global annual total PM_2.5_‐induced premature mortality is three times higher than that from O_3_ mortality, indicating that PM_2.5_ is the primary contributor compared with O_3_. An inter‐comparison of global human health impacts from CEDS, CAMS and ECLIPSEv6b indicates that 80% (CEDS), 81.2% (CAMS), and 77.6% (ECLIPSEv6b) of premature deaths due to anthropogenic activities are associated with Asia and Africa continents.

## Plain Language Summary

In this study, we analyze the differences in emissions of pollutants from three commonly used global anthropogenic emission inventories for the year 2015. We find that there exist large differences among these data sets in terms of global annual total emissions and spatial distributions. By simulating the impact of these emissions on atmospheric chemistry and air quality through a global model, the differences in emissions lead to variations in estimates of particulate matter and ozone levels and their health impacts. Our results show that particulate matter is responsible for three times more premature deaths globally than ozone, with a significant portion of the deaths concentrated in Asia and Africa. This comparison highlights the importance of reducing uncertainties in emission inventories to improve estimates of air pollution and its health impacts.

## Key Points

- This study compared three global anthropogenic emission inventories and their associated air quality and human health impacts
- Large discrepancies of annual total emissions were found among the three anthropogenic emission inventories
- Over 80% of global annual total PM_2.5_‐ and ozone‐induced premature deaths from anthropogenic sources occurred in Asia and Africa

## 1 Introduction

Air pollution plays a major role in the deterioration of our ambient air quality and human health as well as driving the changes of our climate associated with emissions of greenhouse gases and short‐lived climate forcers (SLCFs) (McDuffie et al., 2020). According to the Sixth Assessment Report (AR6) of the Intergovernmental Panel on Climate Change (IPCC), a United Nation organization for assessing the science related to climate change, anthropogenic induced climate change has already impacted weather and climate extremes globally (Huang et al., 2018, 2021; IPCC, 2023; Lund et al., 2023).

Emissions of SLCFs associated with human activities include black carbon (BC), organic carbon (OC), nitrogen oxides (NO_x_ = NO + NO_2_), ammonia (NH_3_), carbon monoxide (CO), sulfur dioxide (SO_2_), and non‐methane volatile organic compounds (NMVOCs), which have altered the composition of the atmosphere and the related fluxes from land and oceanic surfaces (Hoesly et al., 2018; Lund et al., 2020).

Anthropogenic emissions inventories, comprised of comprehensive databases for pollutants and greenhouse gases, are critical for understanding the emissions' impact on the environment and human health using chemistry‐climate models (McDuffie et al., 2020). However, there are limits and discrepancies in emission inventories, which result in uncertainties in quantifying the impact of anthropogenic emissions of chemicals and aerosol precursors on human health. Shami et al. (2022) suggests that emission inventories such as Emissions Database for Global Atmospheric Research (EDGAR), although applicable for continental scale studies, are deemed to having an underestimation when compared to ground observations. Additionally, a significant gap exists in inventories due to the lengthy process of collecting data from all countries, leading to delays in reflecting the most current information in updates (Elguindi et al., 2020; Lund et al., 2023). Difference in process methodology and the data set's coverage, which includes the geographic range and type of emission activities, can also contribute to differences between inventories (Andrew, 2020; Elguindi et al., 2020).

Past studies have used anthropogenic emissions inventories, the Community Emissions Data System (CEDS), the Copernicus Atmosphere Monitoring Service (CAMS) and Evaluating the Climate and Air Quality of Short‐lived Pollutants version 6b (ECLIPSEv6b, hereafter referred to as ECLIPSE) for air quality modeling and analysis. Along with human health impact assessments, emission inventories have been employed to estimate the distribution and magnitude of air pollutants across different regions (Gaubert et al., 2021; Huang et al., 2018, 2021; Jo et al., 2023; Kurokawa & Ohara, 2020; Nawaz et al., 2023; Pino‐Cortés, 2021; Upadhyay et al., 2020; Xiong, Partha, et al., 2022; Xiong et al., 2024). Intercomparison studies have been conducted to analyze the differences between emission inventories; Elguindi et al. (2020) compared top‐down and bottom‐up emissions inventories, finding that annual emissions for species such as CO, BC, and OC were highest in CEDS and lowest in EDGAR for regions like China and India across various pollutants. Kurokawa and Ohara (2020) compared between CEDS and EDGAR, highlighting differences in emissions estimated for pollutants such as NO_x_, CO and PM_2.5_. This is relevant as CAMS is a combination of CEDS and EDGAR, along with other emission data sets. Choulga et al. (2020) compared the global uncertainties between CEDS and EDGAR, where EDGAR's global uncertainty was quantified and CEDS's was not. We would also like to note that ECLIPSE is used by both the CAMS and CEDS emissions inventories. CEDS incorporates ECLIPSE to refine emissions from sectors like gas flaring and shipping (Hoesly et al., 2018), ensuring more accurate data. Similarly, CAMS integrates ECLIPSE (Granier, et al., 2019) to enhance the accuracy of emissions estimates related to specific sectors, such as agricultural waste burning. In this study, we compare three commonly used global emission inventories, including CEDS, CAMS, and ECLIPSE.

Anthropogenic air pollution poses a significant threat to public health, and epidemiological studies have found links between outdoor air pollution and adverse health outcomes, including morbidity and premature deaths associated with respiratory and cardiovascular diseases (e.g., stroke and lung cancer) (Dong et al., 2012; Huang et al., 2020; Lelieveld et al., 2015; Pope, 2000; Turner et al., 2016). It has been estimated that seven million people die each year due to indoor and outdoor air pollution (Sarkodie et al., 2019; World Health Organization, 2024). Even at low exposure levels, long‐term exposure has a considerably greater effect on public health (Beverland et al., 2012; Pozzer et al., 2023). Past studies have reported that global annual premature deaths attributable to ambient long‐term PM_2.5_ (particulate matter with aerodynamic diameters ≤2.5 μm) exposure were estimated to be 4 million in 2015 (Burnett et al., 2018) and numerous studies have documented the impacts of PM_2.5_ on human health (Archer‐Nicholls et al., 2016; Chowdhury et al., 2019; J. Liu et al., 2016). According to the U.S. Environmental Protection Agency (EPA)'s National Ambient Air Quality Standards, the daily maximum 8‐hr average concentration of ground level ozone (O_3_) is set at 0.07 parts per million (ppm) (EPA, 2020). Previous studies have identified the source of air pollution and the human health impacts linked to it for different regions, sectors, and industries (Crippa et al., 2019; Huang et al., 2021; Nawaz et al., 2023; Xiong, Huang, & Du, 2022, Xiong, Partha, et al., 2022). According to Nawaz et al. (2023), the dominant sectors for premature deaths in China are the agricultural and industry, together contributing to 52% of premature deaths for PM_2.5_.

While previous research has explored emissions inventories, none of these studies to our knowledge have specifically examined or compared the disparities in air quality and their resulting impacts on human health arising from the utilization of three distinct global anthropogenic emission inventories and identified the associated drivers. Therefore, this study employed a global chemistry‐climate model, the Community Atmosphere Model with chemistry version 6.0 (CAM‐chem) within the Community Earth System Model version 2.2.0 (CESM2.2) (Danabasoglu et al., 2020) to quantify the effects of global anthropogenic emissions on air quality and human health. We explored the differences between air quality impacts of three commonly used global emissions inventories; CEDS, CAMS, and ECLIPSE (Bran et al., 2022; Huang et al., 2017), and assessed human health impacts estimated from these inventories comparing different regions. This study utilized concentration‐response functions from the Global Burden of Disease 2019 study following Xiong et al. (2024) and Xiong, Partha, et al. (2022).

## 2 Research Methods

### 2.1 Global Anthropogenic Emission Inventories

#### 2.1.1 The Community Emissions Data System (CEDS)

We used the recently updated CEDS data set, CEDS v2021_04_21 (O’Rourke et al., 2021), that contains historical anthropogenic emissions from 1750 to the year 2019 at the horizontal grid resolution of 0.5° latitude by 0.5° longitude, developed at the Pacific Northwest National Laboratory (Hoesly et al., 2018; O’Rourke et al., 2021; Xiong, Partha, et al., 2022). CEDS assesses emissions in 221 different geographic areas, encompassing eight types of fuels and emissions from 55 distinct industry sectors. These industry sectors within CEDS follow the energy statistics guidelines established by the International Energy Agency. CEDS employs a “mosaic” scaling approach to maintain precise information regarding specific fuels and sectors across different data sets, while ensuring consistent methodologies are applied consistently across various locations and time periods. The use of mosaic inventories has become more common as they allow for the use of detailed local emissions while harmonizing this information across broad regional or global scales (Crippa et al., 2019). Sectors of CEDS emission inventory include agricultural, energy, industrial, transportation, residential, solvents, waste, and international shipping, with the global annual total emissions for various species in 2015 being listed in Tables S1 and S2 in Supporting Information S1.

#### 2.1.2 Copernicus Atmosphere Monitoring Service (CAMS)

We have used the most recently updated CAMS data set, CAMS version 5.1 (CAMSv5.1)'s Global Anthropogenic emissions (CAMS‐GLOB‐ANT) (Soulie et al., 2024), a combination of CEDS and EDGAR version 5 (EDGARv5). Both CAMS and EDGAR provide emissions on monthly and annual averages with a resolution of 0.1° × 0.1° (Kuenen et al., 2022; Soulie et al., 2022). CAMS provides anthropogenic emissions of main atmospheric pollutants and greenhouse gases that are relevant in our study such as NO_x_, SO_2_, BC, NH_3_ and NMVOCs and provides emissions for 2000–2022 period. For the NMVOCs emissions, each individual species' emission was calculated separately to find a summation of NMVOCs emitted in the year 2015 (Table S3 in Supporting Information S1). In this paper, we refer to CAMSv5.1's CAMS‐GLOB‐ANT as CAMS. EDGAR methodology for emissions is described in Crippa et al. (2018)and can be found online publicly (https://edgar.jrc.ec.europa.eu/dataset_ap50). CAMS emission inventory has been frequently used in CESM model experiments (Bouarar et al., 2021; Gaubert et al., 2021; Tang et al., 2022, 2023) and in health studies (Bui et al., 2023; Roberts & Wooster, 2021).

#### 2.1.3 Evaluating the Climate and Air Quality Impacts of Short‐Lived Pollutants (ECLIPSE)

We also use global anthropogenic emissions using the International Institute for Applied System Analysis Greenhouse Gas‐Air Pollution Interactions and Synergies (GAINS) (Amann et al., 2011) integrated assessment model, the most recently updated ECLIPSE (Evaluating the Climate and Air Quality Impacts of Short‐Lived Pollutants version 6b) for 2015. All major air pollutants and greenhouse gases from Kyoto Protocol are included in GAINS, with emissions estimates provided for 200 country‐regions (Klimont et al., 2017; Stohl et al., 2015). The species in ECLIPSE, which includes the eight specified pollutants we are focusing on, are at spatial resolutions of 0.5° latitude by 0.5° longitude and is re‐gridded to match the model spatial resolution (Huang et al., 2018). This data set, which includes international shipping, was updated in February 2021. The temporal distribution ranges from 1990 to 2030 in five‐year intervals, 2040, and 2050. This emission inventory includes nine sectors: energy, industry, solvent use, transport, residential combustion, agriculture, open burning of agricultural waste, waste treatment, gas flaring and venting, and international shipping (Table S4 in Supporting Information S1) (Klimont et al., 2025). Previous studies have used ECLIPSE for air quality studies (Huang et al., 2018; Lund et al., 2023; Stohl et al., 2015; Upadhyay et al., 2020) and to estimate the premature deaths due to exposure to PM_2.5_ and O_3_ (Huang et al., 2021).

ECLIPSE provides policy‐driven emission projections based on anticipated regulatory interventions and sectoral activity but does not dynamically adjust for meteorological feedback (Granier, et al., 2019; Klimont et al., 2017; Stohl et al., 2015). However, CAM6‐Chem can simulate how meteorological conditions influence aerosol formation and chemical transformations (Emmons et al., 2020; Tilmes et al., 2019). ECLIPSE emissions estimates indicate that reductions in sulfate oxides (SO_x_) emissions can lead to increased ammonium nitrate (NH_4_NO_3_) formation, contributing to higher secondary inorganic aerosol concentrations. This effect is particularly evident in ammonia‐rich regions like South Asia, where excess NH_3_ shifts toward nitrate aerosol production as sulfate levels decline, altering the chemical composition of atmospheric aerosols (Ge et al., 2023).

In CAM‐chem, secondary organic aerosol (SOA) formation follows a Volatility Basis Set approach, where biogenic and anthropogenic VOCs oxidize via OH, O_3_, and NO_3_ radicals, forming semi‐volatile species that partition onto aerosols (Emmons et al., 2020; Tilmes et al., 2019). The variability in VOC emissions across different inventories affects SOA production, particularly in regions where biomass burning and anthropogenic emissions dominate such as Africa and Asia (Granier, et al., 2019). ECLIPSE projects higher future NH_3_ and NO_x_ emissions in certain regions, particularly in agricultural regions in Southern Asia and Africa, this could lead to enhanced nitrate aerosol formations where SO_4_ levels decline (Klimont et al., 2017).

### 2.2 CESM2 CAM‐Chem Model Simulations

In this study, we used the NSF NCAR (National Center for Atmospheric Research) Community Atmosphere Model version 6.0 with chemistry (CAM‐chem). CAM‐chem is a configuration of CESM2 (Gettelman et al., 2019) and in this study we used CESM2 version 2.2.0. CAM‐chem is coupled with the Community Land Model (CLM) version 5.0 (Iacono et al., 2008; Lawrence et al., 2019). CAM‐chem uses Model for Ozone and Related chemical Tracers as gas‐phase chemical mechanisms to represent the detailed oxidation and reaction pathways for reactive species in the troposphere and stratosphere (Emmons et al., 2020). For aerosol microphysical processes, CAM‐chem uses modal aerosol model with 4 modes (MAM4), which includes Aitken, accumulation, primary carbon and coarse modes (Jo et al., 2023; X. Liu et al., 2016; Tilmes et al., 2019). The aerosol size distributions in each mode of MAM4 are assumed to be lognormal (X. Liu et al., 2016).

The CLM simulates mineral dust emissions using a physically based parameterization driven by surface wind stress, soil properties, and land cover (Leung et al., 2024; Zender et al., 2003). Dust emission fluxes from CLM are then passed to CAM‐chem to account for transport, deposition, and microphysics (X. Liu et al., 2016).

To quantify the impacts of global anthropogenic emissions on air quality modeling, we ran four CAM‐chem simulations from 1 January 2014, to 1 January 2016, at the horizontal resolution of 0.95° latitude by 1.25° longitude, using different anthropogenic emission inventories (Table 1; Text S1 in Supporting Information S1). In our model simulations we use historic global biomass burning emissions for CMIP6 (BB4CMIP) for fire emissions (Van Marle et al., 2017). Biogenic emissions are internally derived using the Model of Emissions of Gases and Aerosols from Nature (Emmons et al., 2020; Guenther et al., 2012; Jo, Tilmes, et al., 2023). CAM‐chem is nudged every 12 hr to the Modern‐Era Retrospective analysis for Research and Applications version 2 meteorological data set. We used the first year of the simulation as spin‐up and the rest for result analysis.

**Table 1:** Model Simulation Configuration for Cases 1–4

Cases	Anthropogenic emission inventory
Case 1	CEDS
Case 2	CAMS
Case 3	ECLIPSE
Case 4	OFF

In Table 1, Cases 1–3 shown represent the simulations with anthropogenic emission inventory from CEDS, CAMS and ECLIPSE, respectively. To quantify the net effect of global anthropogenic emissions, we ran the Case 4, in which we turned off the global anthropogenic emissions, with other model configurations being the same as Cases 1–3. The differences of Cases 1–3 and Case 4 will be the net effects from anthropogenic emissions for CEDS, CAMS and ECLIPSE, respectively.

Before computing mortality rates, the CAM‐chem model's outputs for PM_2.5_ and O_3_ concentrations are downscaled from the original resolution of 0.9° latitude by 1.25° longitude to a finer resolution of 0.1° latitude by 0.1° longitude. This aligns the outputs with the horizontal and gridded resolution of both the total population counts and the country mask. The CAM‐chem simulated PM_2.5_ concentrations underwent downscaling using a recently released satellite‐derived PM_2.5_ product. For CAM‐chem PM_2.5_ outputs, we have downscaled using the newly available satellite data that is at 0.1° latitude x 0.1° longitude resolution. We minimized the negative biases between modeled and observed PM_2.5_ by adjusting the PM_2.5_ concentrations (Van Donkelaar et al., 2021).

### 2.3 Calculations of Human Health Effects: PM_2.5_ and O_3_ Induced Premature Deaths

We used the Global Exposure Mortality Model (GEMM) (Burnett et al., 2018), as described in Huang et al. (2021), to calculate the regional and global annual premature deaths attributable to long‐term ambient surface PM_2.5_ exposure. GEMM predicted non‐accidental deaths from lower respiratory infections (LRIs) and noncommunicable diseases (NCDs) linked to long term exposure to PM_2.5_.The calculation of the Hazard Ratio (HR) function in GEMM is performed for each specific combination of longitude (*i*), latitude (*j*), health endpoint (*h*), and 5‐year age interval group (*a*) at every horizontal model grid box is calculated as expressed below in Equation 1 (Huang et al., 2021).

(1) $$HR_{i,j,h,a}=\exp\left\{\frac{\theta \;\ln\left(\frac{z_{i,j}}{\alpha }+1\right)}{1+\exp^{\left(-\frac{z_{i,j}-\mu }{\upsilon }\right)}}\right\},z_{i,j}=\max\left(0,C_{i,j}-C_{0}\right)$$

θ, μ, α and υ are the GEMM model fit parameters which are obtained from Burnett et al. (2018). We use the standard errors θ to estimate the uncertainty and 95% confidence interval (CI) of PM_2.5_‐induced premature deaths. C_*i*,*j*_ represents the average yearly surface concentration of PM_2.5_ in a specific grid cell denoted by (*i*, *j*). C_0_ is the hypothetical PM_2.5_ concentration set at 2.4 μg/m^3^, below which it is assumed that there is no risk of premature death.

For O_3_‐induced mortality, we followed studies by Huang et al. (2020) and Turner et al. (2016) to calculate the O_3_‐mortality HR function associated with chronic obstructive pulmonary disease (COPD). The study conducted by Turner et al. (2016) links between long‐term exposure to O_3_ and an increased risk for respiratory mortality, and it is found that for every 10 ppb increase in O_3_ levels the respiratory mortality risk is increased by 12%. The O_3_‐mortality HR function in each grid cell (*i*, *j*) for each health endpoint (*h*) and each age group (*a*) is then calculated as:

(2) $${\mathrm{HR}}_{i,j,h,a}=\exp^{\eta \Delta Y_{i,j}},\Delta Y_{i,j}=\max\left(0,\Delta Y_{i,j}-26.7\right)$$

where *η* represents the log‐linear slope between O_3_ concentrations and the HR associated with COPD, with a mean value of 0.0131 (95% CI: 0.0077–0.0191). *Y*_*i*,*j*_ denotes the annual mean Maximum Daily 8‐hr Average (MDA8) O_3_ concentration in grid cell (*i*, *j*). It is assumed that the threshold value of MDA8 O_3_ concentration is 26.7 ppb, below which there is no mortality risk associated with O_3_‐induced COPD (Huang et al., 2021; Turner et al., 2016).

The CAM‐chem model simulation outputs are regridded to 0.1° latitude and 0.1° longitude from their 0.9° latitude x 1.25° longitude resolution, which is required for the country mask and gridded total population count. To assess impact on human health caused by exposure to ambient PM_2.5_ and O_3_ we consider the baseline mortality rate (BMR) from the latest Global Burden of Disease 2019 (hereafter referred to GBD 2019) (GBD 2019 Risk Factors Collaborators, 2020), the gridded population density (POP), and the HR as shown in Equation 3:

(3) Mi,j,h,a=POPi,j,a×BMRi,j,h,a×HRi,j,h,a−1HRi,j,h,a

where *M*_*i*,*j*,*h*,*a*_ is the gridded annual total premature deaths (APDs) associated with PM_2.5_ and O_3_ exposure at each grid box (*i*, *j*) for each health endpoint (*h*) and age group (*a*); POP_*i*,*j*,*a*_ denotes gridded POP which is estimated as the product of population counts in each grid cell (*i*, *j*) and the age specific (5 years interval) population fraction.

Following Huang et al. (2021), the gridded POP is sourced from the Gridded Population of the World version 4 for the year 2015 (Center for International Earth Science Information Network – CIESIN – Columbia University, 2018) at a horizontal resolution of 2.5 min × 2.5 min. This data is then regridded to a resolution of 0.1° latitude × 0.1° longitude.

The Years of Lives Lost (YLL), for each grid cell (*i*, *j*), health endpoint (*h*) and age group (*a*) are calculated in Equation 4 as:

(4) $${\text{YLL}}_{i,j,h,a}=M_{i,j,h,a}\times {\text{MYLL}}_{i,j,h,a}$$

MYLL_*i*,*j*,*h*,*a*_ is the mean YLL for each health point and each age group attributed to all causes from GBD 2019 for the year 2015.

Following previous studies (Huang et al., 2021; Xiong, Partha, et al., 2022), we divide the globe into 11 regions based on the classification by the International Monetary Fund (IMF): China, India, United States of America (USA), Canada, Eastern and Central Europe (ECEUROPE), Western Europe (WEUROPE), Latin America (LATIN), North Africa and Middle East (NAME), Rest of Asia (ROA), Sub‐Saharan Africa (SSA) and Rest of the world (ROW). We calculated the regional and global annual health burdens of exposure to PM_2.5_ and O_3_ by adding up each grid cell for each simulation (Huang et al., 2020, 2021). We subtracted all total mortalities in each simulation from Cases 1–3 from Case 4 to quantify the net health effects for each global anthropogenic emission inventory (CEDS, CAMS and ECLIPSE).

## 3 Results

### 3.1 Intercomparisons of Regional and Global Spatial Patterns of Emissions Among CEDS, CAMS and ECLIPSE for the Year 2015

Table 2 lists the annual total emissions of BC, OC, NO_2_, CO, NMVOC, NH_3_, and SO_2_, respectively, from CEDS, CAMS and ECLIPSE for the year 2015. The total emissions from the CAMS and ECLIPSE inventories were then compared to the total emissions using CEDS as baseline. In general, global annual total emissions of NO_2_ and CO for the year 2015 are consistent among CEDS, CAMS and ECLIPSE (Table 2).

**Table 2:** Global Annual Total Emissions of Various Species for the Year 2015 From CEDS, CAMS and ECLIPSE

	BC	OC	NO_2_	CO	NMVOC	NH_3_	SO_2_
CEDS	6.0	14.0	121.9	565.6	149.6	59.4	92.1
CAMS	4. 8	16.5	121.5	578.1	148.3	49.1	99.7
ECLIPSE	6.4	13.9	125.0	548.4	112.0	61.0	73.4

*Note.* Units: Tg specie per year. In CAMS inventory, NO_x_ was archived as NO, and we converted it to NO_2_ for comparing with CEDS and ECLIPSE.

**Figure 1:**
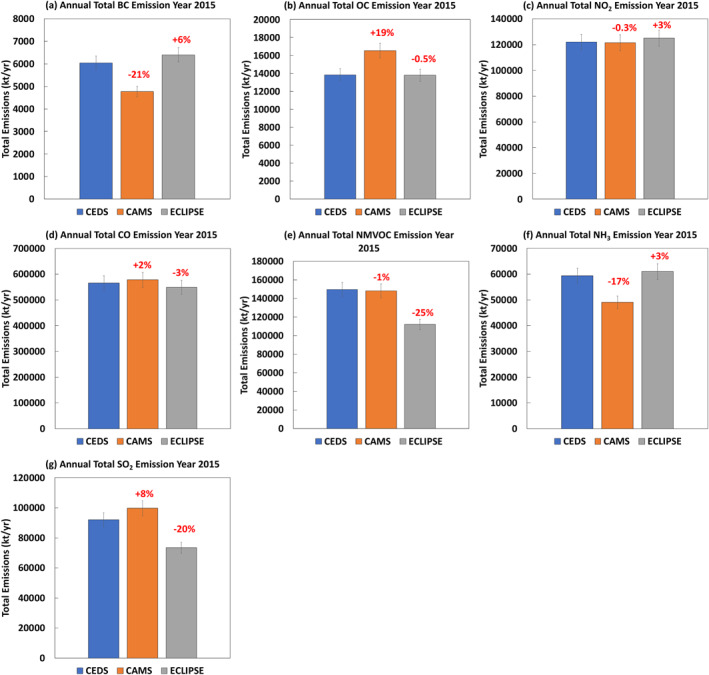
An inter‐comparison of global annual total emissions of (a) black carbon, (b) organic carbon, (c) NO_2_, (d) CO, (e) non‐methane volatile organic compound, (f) NH_3_, and (g) SO_2_ for the year 2015 from Community Emissions Data System (blue), Copernicus Atmosphere Monitoring Service (orange) and Evaluating of the Climate and Air Quality Impacts of Short‐Lived Pollutants (gray). Units: kt specie/yr.

In terms of O_3_ precursors such as CO, we discovered that CO accounted for 56% (CEDS), 57% (CAMS), and 58% (ECLIPSE) of the respective global annual total anthropogenic emissions (Figure 1) in 2015 (Figure 1d). The annual total emissions of NMVOC between CEDS (149.6 Tg) and CAMS (148.3 Tg) were also consistent, with values from ECLIPSE for NMVOC 25% lower than that from CEDS (Figure 1e) (Placet et al., 2000). Large discrepancies associated with global annual total emissions of NH_3_ in 2015 between CEDS and CAMS were found, with the latter 20% lower than the former (Figure 1f). In terms of PM_2.5_ precursors we find that globally the annual total of BC emissions were 6.0 (CEDS), 4.8 (CAMS) and 6.4 Tg/yr (ECLIPSE), while OC was 13.9 (CEDS), 16.5 (CAMS) and 13.8 Tg/yr (ECLIPSE) (Contini et al., 2018). Compared with CEDS, the global annual total emissions of BC from CAMS and ECLIPSE are 21% lower and 6% higher, respectively (Figure 1a). For OC, the global annual total emissions between CEDS (14.0 Tg) and ECLIPSE (13.9 Tg) in 2015 were comparable. However, we found that the global annual total OC emissions for the year 2015 from CAMS were about 20% higher than that from CEDS (Figure 1b). Nevertheless, NH_3_ emissions from CEDS and ECLIPSE were consistent. Global annual total emissions of SO_2_ from CAMS and ECLIPSE for the year 2015 were 8% higher, and 20% lower than that from CEDS, respectively (Figure 1g).

Previously, Hoesly et al. (2018) found that CEDS estimates were generally larger than those of EDGARv4.3 for global estimates across most species. In our comparison, we found that the global annual total emissions of BC and NH_3_ from CEDS (Tables S1 and S2 in Supporting Information S1) in 2015 are much higher than those from CAMS (Table S3 in Supporting Information S1), which could be attributable to differences in sector distributions (Hoesly et al., 2018; Kuenen et al., 2022).

### 3.2 Validations of Model Simulations Against Observations

**Figure 2:**
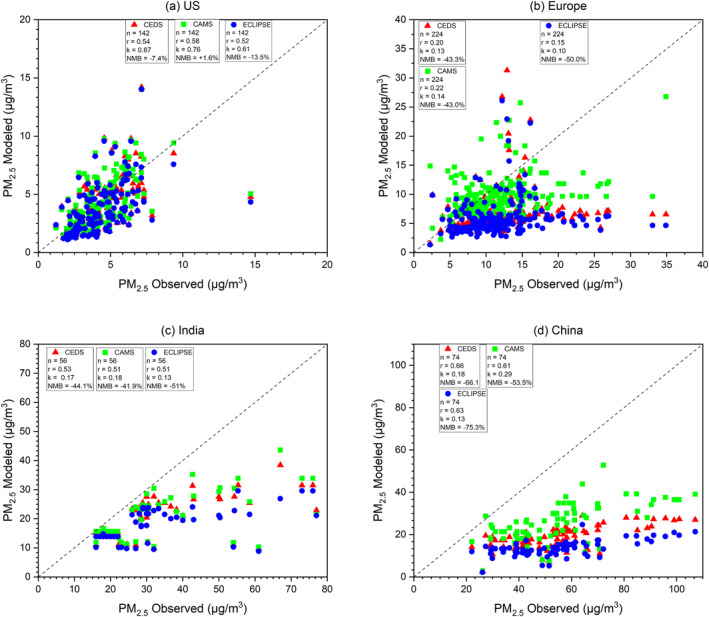
Scatter plot of observed and model surface PM_2.5_ concentrations; Community Emissions Data System (red triangle), Copernicus Atmosphere Monitoring Service (green square), and Evaluating of the Climate and Air Quality Impacts of Short‐Lived Pollutants (blue circle) values over (a) United States, (b) Europe, (c) India, and (d) China in 2015. The dashed line is 1:1 reference line.

To validate our model simulation, we employed surface PM_2.5_ measurement networks from the US, China, India, and Europe. Subsequently, we compared these measurements with the results of our CAM‐chem model simulations for the year 2015. Following the methodology of Huang et al. (2021) and Xiong, Partha, et al. (2022), we calculated the annual average surface PM_2.5_ concentrations. Measurements from the US, Europe, India, and China were used in these assessments. We specifically obtained detailed PM_2.5_ annual concentration data for the United States (US) from the Improved Air Quality System network, downloaded from the EPA's Air Quality System (AQS) Data Mart (https://aqs.epa.gov/aqsweb/airdata/download_files.html). Annual PM_2.5_ concentrations from India were collected from the National Ambient Air Quality Monitoring Program (https://cpcb.nic.in/namp‐data/). We have also used annual PM_2.5_ concentration data from the European Environment Agency's Air Quality e‐Reporting system for Europe (EEA; https://www.eea.europa.eu/en/analysis/maps‐and‐charts/air‐quality‐statistics‐dashboards). A total of 142, 224, 56, and 74 ground stations were chosen in US (Figure 2a), Europe (Figure 2b), India (Figure 2c), and China (Figure 2d), respectively.

To compare observed data and the model's PM_2.5_ simulation in the four different regions, we used statistical techniques such as normalized mean bias (NMB) and Pearson's correlation coefficient (*r*).

When validated against ground observational data, the NMB values calculated for CEDS, CAMS, and ECLIPSE reveal distinct patterns across various regions. Across Europe, all three emission inventories consistently underestimate surface PM_2.5_ concentrations with NMB values of −43.3% (CEDS), −43% (CAMS), and −50% (ECLIPSE). Similar underestimations are observed in India (CEDS: −44.1%, CAMS: −41.9%, ECLIPSE: −51.0%) and China (CEDS: −66.1%, CAMS: −53.5%, ECLIPSE: −75.3%). In contrast, the models perform better over the US, with NMB values of −7.4% (CEDS), +1.6% (CAMS), and −13.5% (ECLIPSE), indicating relatively minor biases. ECLIPSE exhibits the largest negative biases across Europe, India, and China, although in the US, it shows a moderate underestimation compared to CEDS and CAMS. Among the three inventories, CAMS demonstrates the least bias overall, with smaller under‐ and overestimations across all regions.

CESM2 underestimates surface mineral dust emissions over source regions (Leung et al., 2025). This underestimation is especially pronounced in regions such as East Asia, where observed dust levels are significantly higher than those simulated (Wu et al., 2019). This uncertainty may partially contribute to the underestimates of model simulated surface PM_2.5_ concentrations, compared with observations.

In an earlier version of the CAM‐chem model simulation, CAM5‐Chem using the ECLIPSE V5a inventory in Huang et al. (2021) observed that surface PM_2.5_ concentrations attributed to higher SOA levels compared to observations (Tsigaridis et al., 2014). Similar overestimations persist in CAM6‐chem as identified by J. Liu et al. (2020) and Y. Liu et al. (2020). Another likely reason for overestimation can also be due to the summertime overestimates of sulfate concentrations (Tilmes et al., 2015), thereby compounding the overall PM_2.5_ overestimation. Consistent with these findings, Xiong, Partha, et al. (2022) reported NMB values of −57.7%, −67.7%, and −44.4% for CEDS over Europe, China and India, respectively.

As shown in Section 3.1 (Figures 1c, 1e, and 1g), precursors linked with formation of PM_2.5_, such as NO_2_, NMVOCs, NH_3_, and SO_2_, are much lower in ECLIPSE (except for NO_2_) compared to CEDS and CAMS. Consistently, the relative difference of NMB for CEDS when compared to ECLIPSE is −81% lower, while for CAMS the NMB relative difference is +78% higher. The differences in the different emission inventories and their impact on aerosol formation critically influence the representation of aerosols in climate models (Jo et al., 2021).

**Figure 3:**
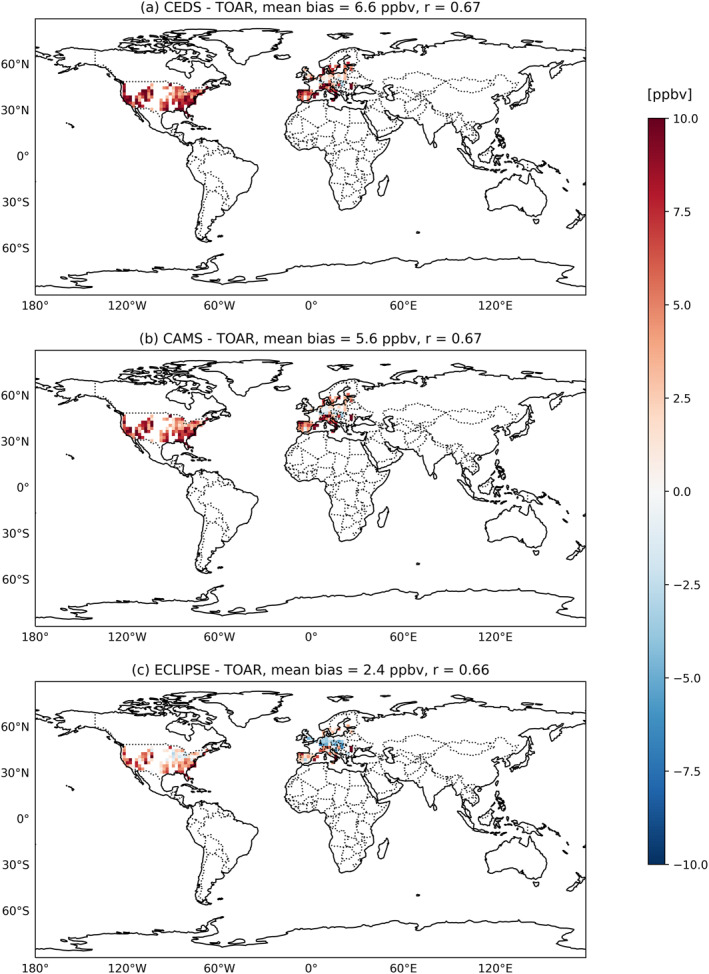
The comparison between model simulations of surface O_3_ concentrations using the Community Emissions Data System, Copernicus Atmosphere Monitoring Service and Evaluating of the Climate and Air Quality Impacts of Short‐Lived Pollutants emissions inventories and Tropospheric Ozone Assessment Report with (a) CEDS‐TOAR, (b) CAMS‐TOAR, and (c) ECLIPSE‐TOAR. The average mean bias is determined as the mean variation in each grid cell when both observations and simulations are available for the year of 2015. Unfilled/White grid cells indicate areas without recorded measurements.

To verify the accuracy of CAM‐chem simulated O_3_ concentration, we employed the surface O_3_ concentrations from the Tropospheric Ozone Assessment Report (TOAR) II (Huang et al., 2021; Xiong, Partha, et al., 2022). This involved regridding the model from 0.9° × 1.25° longitude‐latitude grid to 2° × 2° latitude‐longitude grid and comparing it with TOAR. We have used an updated TOAR data, TOAR II, which includes 2015 (Fiore et al., 2009; Schultz et al., 2017; Young et al., 2013, 2018), allowing for more direct comparisons. The generated data set focused on rural observational data and focused on surface O_3_ observations. There were notable discrepancies between the surface O_3_ concentrations predicted by the model simulations and the actual observed values reported in the TOAR data, as illustrated in Figure 3. We found that the model overestimated surface O_3_ concentrations compared with TOAR observations, with mean biases of 6.6 ppbv for CEDS (Figure 3a), 5.6 ppbv for CAMS (Figure 3b), and 2.4 ppbv for ECLIPSE (Figure 3c), respectively. The *r* coefficients were 0.67, 0.67, and 0.66 for CEDS, CAMS and ECLIPSE respectively. Previous studies have reported similar overestimations (Huang et al., 2021; Lamarque et al., 2012; Tilmes et al., 2015).

It is important to note that the Huang et al. (2021) study used an earlier version of the ECLIPSE emissions inventory (ECLIPSEv5a), whereas our study utilizes the updated inventory (ECLIPSEv6b). As a result, our reported mean bias (2.4 ppbv) is substantially lower than that reported by Huang et al. (2021), which was 17.9 ppbv. One of the reasons for the differences with the updated ECLIPSE emissions inventory could be due to the revised international shipping, updates for several sectors such as power plants, flaring, transportation industry‐all of which are sources for NMVOCs and NO_x_, which contribute to the formation of O_3_. Another reason is that there was an update on the general legislation and historical data especially for the year 2015 (https://iiasa.ac.at/models‐tools‐data/global‐emission‐fields‐of‐air‐pollutants‐and‐ghgs).

We recognize that our simulations using CEDS and CAMS emissions inventories exhibit high biases in surface O_3_ concentrations. Consequently, this would impact the estimated premature deaths due to anthropogenic activities associated with chronic obstructive pulmonary disease (COPD) attributable to long‐term surface O_3_ exposure. Our simulation using ECLIPSE emissions inventory demonstrated a significantly lower mean bias when compared to a previous study (Huang et al., 2021) indicating an improvement. Lastly, it's important to highlight that many regions lack recorded observational data, resulting in unfilled or blank grid cells on the maps.

### 3.3 Air Quality Impacts

In the following section, we examined the impact of the three anthropogenic emissions inventories on global ambient surface concentrations of O_3_, NO_x_, and PM_2.5_ respectively for the year 2015, while subtracting the Case 4 from each of the simulations with different emission inventories. Other details, including changes in BC, CO, NMVOC, NH_3_ and SO_2_ are provided in Text S2 in Supporting Information S1.

**Figure 4:**
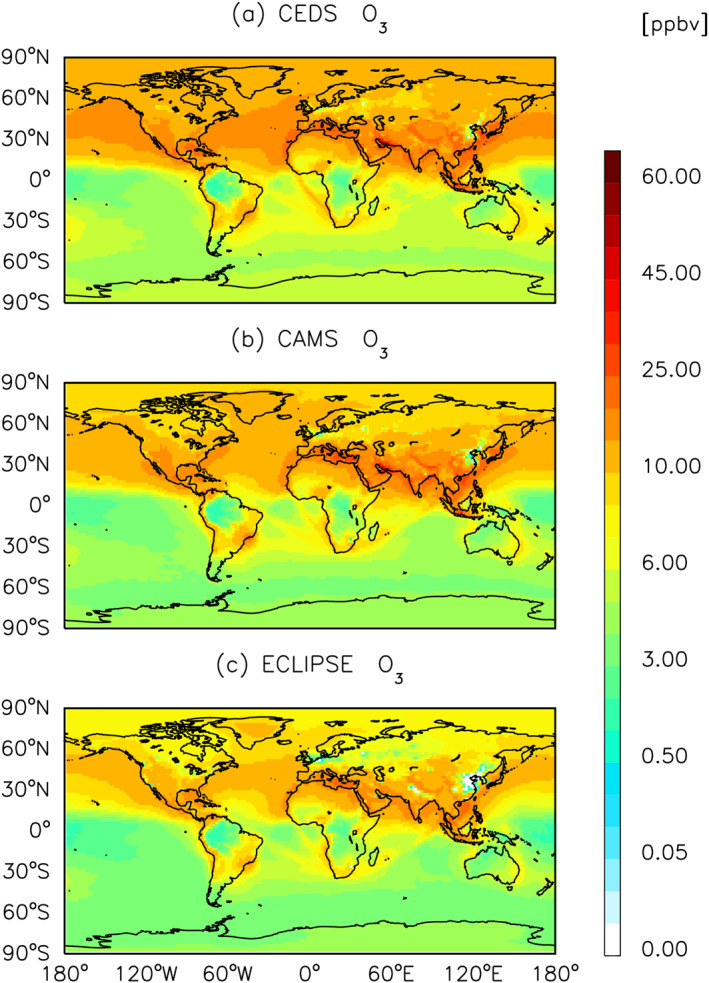
Global annual mean surface O_3_ concentrations for the year 2015 from CESM2.2 CAM‐chem, with anthropogenic emission inventories of (a) CEDS, (b) CAMS, and (c) ECLIPSE.

**Figure 5:**
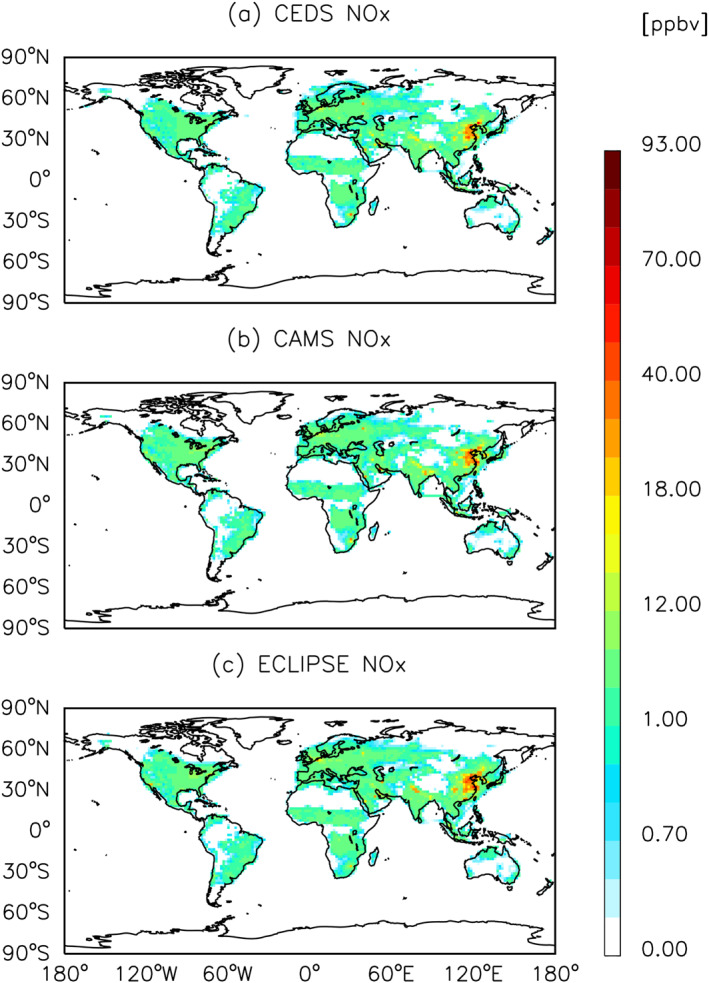
Same as Figure 4 but for NO_x_.

**Figure 6:**
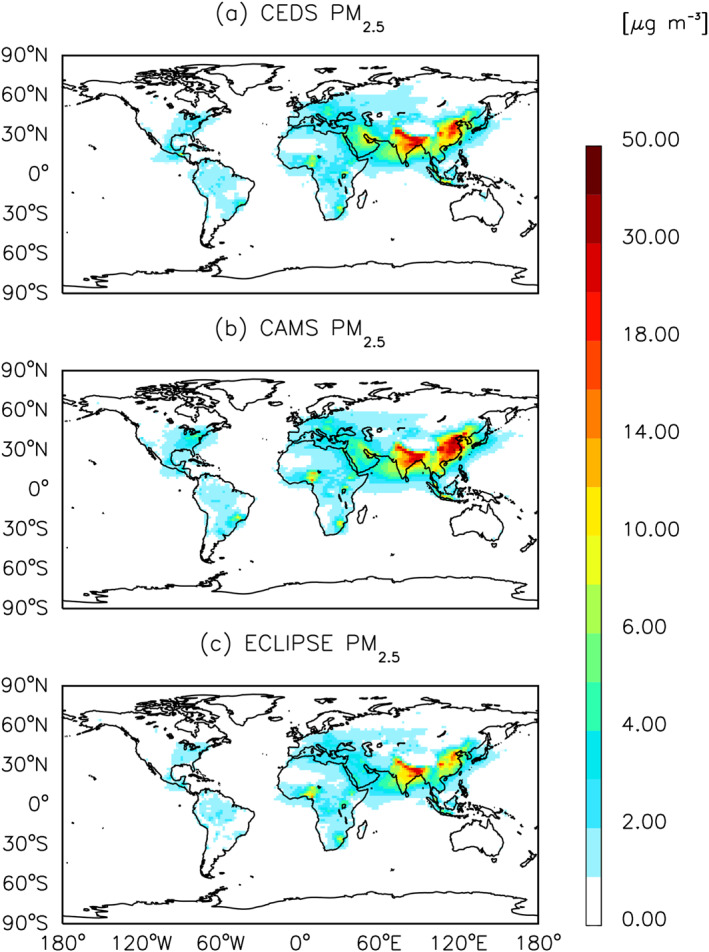
Same as Figure 4 but for PM_2.5_.

Overall, a majority of CEDS emission sources come from Asia, Europe, and North America. Similar spatial patterns are found for ECLIPSE and CAMS (Figures 4, 5, 6 and Figures S2–S6 in Supporting Information S1). However, the spatial differences in pollutants such as O_3_ and PM_2.5_ are more substantial between CEDS and ECLIPSE than between CEDS and CAMS.

Figures 4, 5, 6 show the global spatial distribution of annual average concentration of O_3_, NO_x_ and PM_2.5_ for the year 2015 (see Figures S2–S6 in Supporting Information S1 for BC, CO, NMVOC, NH_3_ and SO_2_ species).

NMVOCs and NO_x_ are the primary precursors leading to net O_3_ production (McDuffie et al., 2020). The global annual mean surface O_3_ concentrations driven by anthropogenic activities are 9.3, 8.1, and 6.7 ppbv for CEDS, CAMS and ECLIPSE respectively. For surface O_3_ impacts, we found that annual surface O_3_ concentrations are highest over Asia, Northern Africa, and the western coast of North America (Figure 4). The global spatial variability of surface O_3_ concentrations is primarily driven by meteorological conditions rather than the spatial distribution of its precursors, such as NO_x_, due to its longer atmospheric lifetime (Ansari et al., 2016). When examining the distribution of NO_x_ across all three inventories (Figure 5), the patterns appear comparable, with global mean concentrations of 0.4, 0.4, and 0.5 ppbv for CEDS, CAMS, and ECLIPSE respectively. A higher NO_x_ concentration was consistently observed in Eastern Asia across three inventories.

The three simulations revealed a similar distribution of surface PM_2.5_, with higher concentrations in Asia, Africa, South America, North America, and Australia (Figure 6).We found that annual mean surface PM_2.5_ concentrations, driven by anthropogenic activities, exhibited substantial burdens over India at 13.8 (CEDS), 14.2 (CAMS), and 11.1 (ECLIPSE) μg/m^3^, and over China at 5.5 (CEDS), 8.0 (CAMS), and 3.9 (ECLIPSE) μg/m^3^ (Table S5 in Supporting Information S1). India contributes on average 43% of total PM_2.5_ concentrations attributable to anthropogenic activities across the three inventories. Large surface impacts of BC impacts are observed over South Asia, Eastern Asia, Africa, and South America (Figure S2 in Supporting Information S1), which are consistent with previous studies (Huang et al., 2018, 2020, 2021). For CO (Figure S3 in Supporting Information S1), we find that the annual global mean surface concentration for CEDS, CAMS, and ECLIPSE majority of the impacts are found in Eastern Asia. The spatial distributions of NMVOCs (Figure S4 in Supporting Information S1) and NH_3_ (Figure S5 in Supporting Information S1) are similar across all inventories, with elevated concentrations in South America, Africa, and Eastern Asia. ECLIPSE's simulated concentrations for NMVOCs are lower than CAMS and CEDS, averaging 3.1 ppbv annually. In all three simulations, SO_2_ (Figure S6 in Supporting Information S1) concentrations are predominantly over Asia (Eastern and Southern), with CAMS showing higher impacts in Eastern Asia. The differences in spatial distributions of global annual mean surface PM_2.5_ and O_3_ can be found in Figures S7 and S8 in Supporting Information S1.

### 3.4 Impacts on Human Health

**Figure 7:**
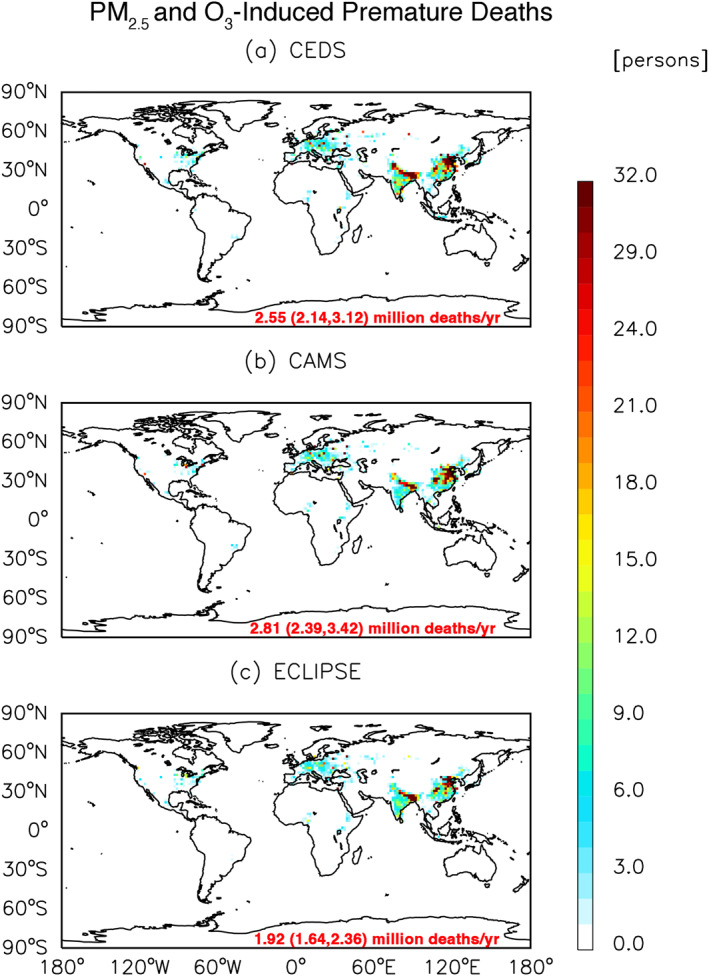
Global annual total PM_2.5_‐ and O_3_‐induced premature deaths due to anthropogenic air pollution sources for the year 2015, for (a) Community Emissions Data System, (b) Copernicus Atmosphere Monitoring Service and (c) Evaluating of the Climate and Air Quality Impacts of Short‐Lived Pollutants, respectively. Parenthesis shows the uncertainty and 95% confidence intervals.

**Figure 8:**
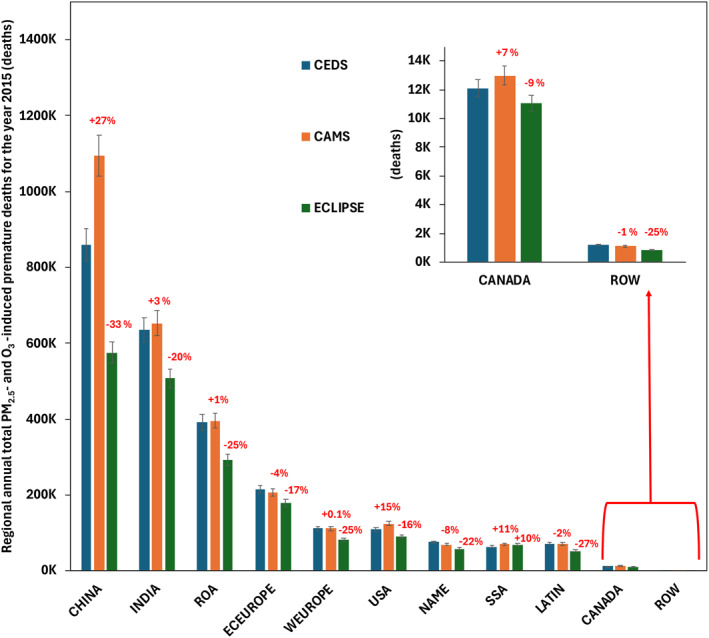
Combined regional annual total PM_2.5_‐ and O_3_‐induced premature deaths for the year 2015 for Community Emissions Data System (CEDS) (blue), Copernicus Atmosphere Monitoring Service (CAMS) (orange) and Evaluating of the Climate and Air Quality Impacts of Short‐Lived Pollutants (ECLIPSE) (green). Percentages in the bar plots are the percentage differences for CAMS and ECLIPSE using CEDS as a baseline. The error bars represent 95% confidence intervals.

Global APDs following Huang et al. (2021) methodology is provided in Table 3. The estimated global PM_2.5_‐ and O_3_‐induced APDs attributed to anthropogenic activities for CEDS, CAMS and ECLIPSE are 2.5 (95% CI: 2.1–3.1), 2.8 (95% CI: 2.4–3.4), and 1.9 (95% CI: 1.6–2.4) million (Figure 7). Regionally, PM_2.5_‐ and O_3_‐induced mortalities from anthropogenic activities peak in China, India, and ROA (Figure 8). On average, China represents 33.8%, 38.9%, and 30.0% of the global total premature mortality in CEDS, CAMS, and ECLIPSE, respectively. For India, these figures are 24.9%, 23.2%, and 26.4% across the three inventories, while ROA accounts for 15.4%, 14.1%, and 15.2% in CEDS, CAMS, and ECLIPSE, respectively.

**Table 3:** Global Annual Total PM_2.5_‐ and O_3_‐Induced Premature Deaths and Years of Life Lost in 2015

Cases	Species	2015	
		Premature deaths (x1,000 persons)	Years of lives lost (10^6^ years)
CEDS	O_3_	695.2 (445.7, 948.0)	11.7 (6.6, 15.6)
	PM_2.5_	1852.0 (1698.1, 2142.7)	43.6 (41.8, 48.4)
	PM_2.5_ + O_3_	2547.3 (2142.7, 3115.3)	55.4 (50.6, 64.1)
CAMS	O_3_	670.6 (429.1, 914.7)	11.4 (7.3, 15.1)
	PM_2.5_	2140.7 (1964.7, 2500.5)	51.4 (47.2, 55.4)
	PM_2.5_ + O_3_	2811.3 (2393.8, 3415.2)	62.7 (54.5, 70.6)
ECLIPSE	O_3_	422.4 (263.4, 599.8)	25.6 (11.7, 46.0)
	PM_2.5_	1499.7 (1374.0, 1755.2)	36.5 (33.4, 39.4)
	PM_2.5_ + O_3_	1922.1 (1637.3, 2355.0)	62.1 (45.2, 85.4)

*Note.* The parenthesis denotes the 95% confidence intervals.

When comparing between the inventories' PM_2.5_‐induced APDs, we use CEDS as the baseline for comparisons. Regionally we find that China and US have the highest percentage discrepancy compared to CAMS, with an average difference of +41.5% for China and +22.8% for US (Table S6 in Supporting Information S1). In CAMS, we find that the NAME region has the lowest PM_2.5_ APDs percentage difference at −10.8%. ECLIPSE generally showed lower percentage differences, with notable values in regions like China (−23.9%), Eastern Europe (−15.9%), Latin America (−28.0%), and Western Europe (−23.5%). However, SSA showed a positive percentage difference (+13.6%) for ECLIPSE.

Regionally, when comparing the O_3_‐induced deaths from the three inventories, we observe a discernible pattern for ECLIPSE, as the percentage differences for O_3_ APDs (Table S7 in Supporting Information S1) are significantly larger compared to the baseline, with China showing the highest difference at −57.0% and the ROW region coming in second at −48.1%. ECLIPSE has the lowest overall total O_3_ APDs across all regions when compared to the baseline. For CAMS, the percentage difference for all regions is less than 10%. Both ECLIPSE and CAMS show a significant percentage difference in the ROW region, at −29% and −6%, respectively. Please refer to Tables S6 and S7 in Supporting Information S1 for detailed APD differences between the three emissions inventories.

YLL associated with PM_2.5_ + O_3_ in 2015 were calculated to be 55.4 (95% CI: 50.6–64.1), 62.7 (95% CI: 54.5–70.6) and 62.1 (95% CI: 45.2–85.4) million years CEDS, CAMS, and ECLIPSE, respectively. The YLLs (Table 3) for CAMS are significantly higher than the baseline, and this trend is also seen in the APDs. On a regional scale we find that China, India an ROA have the largest PM_2.5_ mortalities in 2015, similarly the case for O_3_‐induced mortalities. However, we notice an increase in the mortalities for PM_2.5_ for the CAMS inventory, with significance in China, USA, and Canada at +42%, +23% and +12% percentage difference. Consistently, ECLIPSE has the largest percentage difference across all regions, with a much lower global annual PM_2.5_‐induced deaths‐the largest difference being in LATIN region at −28% followed by China at −24% difference.

Following Xiong, Partha, et al. (2022), we investigated the impact the economic disparities would have on mortality due to PM_2.5_ and O_3_. We had differentiated our data between developing and developed nations using data from the IMF. In this study we have found that developing nations have a higher PM_2.5_‐ and O_3_‐induced mortalities per capita (×10^5^) than developed nations (Table 4). For 2015, we have estimated that the PM_2.5_‐ and O_3_‐induced mortalities global APD rate per capita (×10^5^) is 2.3 (95% CI: 1.9–2.8), 2.5 (95% CI: 2.1–3.0), and 1.9 (95% CI: 1.5–2.2) per 100,000 for CEDS, CAMS and ECLIPSE respectively in developed nations. In developing nations, the global PM_2.5_ + O_3_ APD rates per 100,000 are 23.1 (95% CI: 19.5–28.5) for CEDS, 25.6 (95% CI: 23.0–31.2) for CAMS, and 17.4 (95% CI: 14.8–21.2) for ECLIPSE.

**Table 4:** Comparisons of PM_2.5_‐ and O_3_‐Induced Premature Death (APD) Rates (per 100,000 Population) Between Developed and Developing Countries in 2015

Cases	Species	Regions	2015 APD rates
CEDS	O_3_	Developed	0.74 (0.46, 1.0)
		Developing	6.2 (4.0, 8.5)
	PM_2.5_	Developed	1.6 (1.5, 1.8)
		Developing	16.9 (15.5, 19.9)
	PM_2.5_ + O_3_	Developed	2.3 (1.9, 2.8)
		Developing	23.1 (19.5, 28.5)
CAMS	O_3_	Developed	0.70 (0.43, 0.96)
		Developing	6.0 (3.9, 8.2)
	PM_2.5_	Developed	1.6 (1.5, 1.8)
		Developing	19.6 (18.0, 23.0)
	PM_2.5_ + O_3_	Developed	2.5 (2.1, 3.0)
		Developing	25.6 (23.0, 31.2)
ECLIPSE	O_3_	Developed	0.54 (0.32, 0.72)
		Developing	3.7 (2.3, 5.3)
	PM_2.5_	Developed	1.3 (1.2, 1.5)
		Developing	13.7 (12.5, 16.1)
	PM_2.5_ + O_3_	Developed	1.9 (1.5, 2.2)
		Developing	17.4 (14.8, 21.2)

*Note.* Parenthesis shows the uncertainty and 95% confidence intervals. Developed countries include the USA, Canada, WEurope, and ROW; while developing countries refer to China, India, ROA, SSA, ECEurope, NAME and LATIN.

Our study reveals that in developed nations, the ECLIPSE PM_2.5_ + O_3_ associated APD rate is, on average, 20.4% lower than that of CEDS, while the CAMS APD rate is 7.2% higher than that of CEDS. We found that the PM_2.5_ + O_3_ APD rate in developing nations on average is 11 times greater than those in developed nations across the three inventories; at 9.9 (CEDS), 10.2 (CAMS), and 9.3 (ECLIPSE). Additionally, when comparing with CEDS as the baseline, ECLIPSE is, on average, 17.2% lower, while CAMS is 12.9% higher.

### 3.5 Discussion

We have employed a state‐of‐the‐art chemistry‐climate model, CAM‐chem to quantify the impacts of anthropogenic emission on surface air quality and the associated premature deaths for the year 2015 based on three commonly used emissions inventories CEDS, CAMS and ECLIPSE.

**Figure 9:**
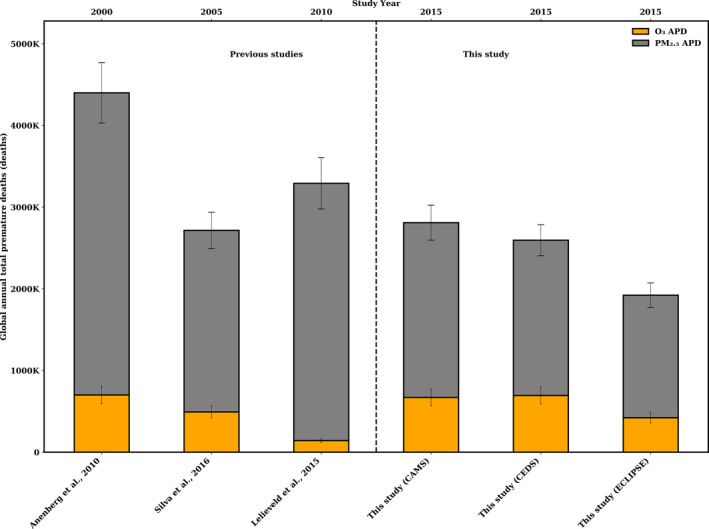
Comparisons of global total annual total premature deaths (APDs) associated with anthropogenic sources, APD associated with O_3_ (orange), and APD associated with PM_2.5_ (gray). Total APD is including both PM_2.5_ + O_3_. The error bars show 95% confidence intervals of estimated premature deaths resulted from PM_2.5_ + O_3_ exposures.

**Figure 10:**
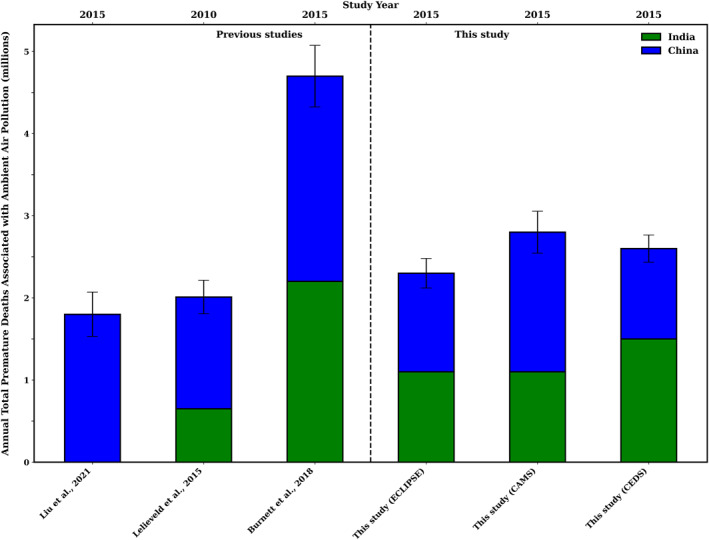
Comparisons of global annual total premature deaths associated with ambient PM_2.5_ for India and China. India and China are represented by green and blue bars respectively. The *y* axis is in millions. The error bars show 95% confidence intervals of estimated premature deaths.

Figures 9 and 10 provide a visual representation of previous studies in comparison to this study. For 2015, our global estimate of O_3_ related mortality due to COPD was 695,000 (95% CI: 445,000–948,000), 671,000 (95% CI: 429,000–915,000), 422,000 (95% CI: 263,000–600,000) deaths for CEDS, CAMS and ECLIPSE, respectively (Figure 9).

The estimations using the CEDS and CAMS emissions inventory agree with the 700,000 deaths reported by Anenberg et al. (2010) associated with O_3_ exposure, which utilized EDGAR emissions inventory (Horowitz, 2006), for the year 2000 (Figure 9). Similarly, a study by Lelieveld et al. (2015) also used EDGAR emissions for their 2010 analysis. They reported global annual 142,00 deaths in 2010 which is significantly lower (Figure 9). While a study by Silva et al. (2016), using the Representative Concentration Pathway 8.5 (RCP 8.5) emissions inventory based on EDGAR data, reported 470,000 deaths in 2005 which is closer to our estimate using ECLIPSE emissions inventory (Figure 9).

Furthermore, our estimates for global PM_2.5_‐induced premature deaths due to LRI and NCD in 2015 were 1.8 (95% CI: 1.7–2.1), 2.1 (95% CI: 2.0–2.5), 1.5 (95% CI: 1.4–1.8) million for CEDS, CAMS and ECLIPSE, respectively (Figure 9). Our global estimation of PM_2.5_‐induced deaths for CEDS and CAMS agrees with Silva et al. (2016), which estimated 2.2 (95% CI: 1.0–3.3) million APDs for 2005. However, study by Anenberg et al. (2010) estimated 3.7 million APDs attributable to anthropogenic PM_2.5_, which is significantly higher than all three of our estimates (Figure 9).

Previous studies have also estimated PM_2.5_ + O_3_ anthropogenic APDs as 2.7 (Silva et al., 2016), and 3.3 (Lelieveld et al., 2015) million for 2005 and 2010 respectively, which are close to the APDs estimations in this study at 2.5 (95% CI: 2.1–3.1), 2.8 (95% CI: 2.3–3.4), and 1.9 (95% CI: 1.6–2.3) million for CEDS, CAMS and ECLIPSE, respectively (Figure 9).

Regionally, premature deaths associated with anthropogenic PM_2.5_ + O_3_ were documented in China to be 1.2 million (Lelieveld et al., 2015) in 2010 (Figure 10). In comparison, our study has estimated 0.9 (95% CI: 0.7–1.1), 1.1 (95% CI: 0.9–1.3) and 0.6 (95% CI: 0.5–0.7) million deaths for CEDS, CAMS and ECLIPSE, respectively (Figure 10). This study estimates APDs due to anthropogenic PM_2.5_ + O_3_ in India to be 0.6 (95% CI: 0.5–0.8), 0.7 (95% CI: 0.5–0.8), and 0.5 million (95% CI: 0.4–0.7) in 2015 for CEDS, CAMS, and ECLIPSE, respectively. These estimates are similar to those reported by Lelieveld et al. (2015), who estimated APDs associated with anthropogenic PM_2.5_ + O_3_ in India to be 0.6 million in 2010 (Figure 10).

Previous study conducted by Lelieveld et al. (2015) estimated that, when exposure to anthropogenic PM_2.5_ + O_3,_ India and China accounted for 41% and 19% of total APD, respectively. Similarly, Silva et al. (2016) found that in 2005, exposure to PM_2.5_ + O_3_ for the year 2005 resulted in most of the mortality occurring in East Asia (45%) and India (20%).

The results of our study are consistent with previous studies when investigating both global natural and anthropogenic emissions. We found that the 6.8 million (95% CI: 6.0–7.6) APD reported by Xiong, Partha, et al. (2022) in 2015, using the CEDS emissions inventory were, while higher, comparable to our estimates of global premature deaths attributable to PM_2.5_ + O_3_ exposure. Our estimates were 5.3 (95% CI: 4.6–6.4), 5.6 (95% CI: 4.9–6.7), and 4.7 (95% CI: 4.1–5.6) million for CEDS, CAMS and ECLIPSE respectively (Figure S8 in Supporting Information S1). However, previous studies reported significantly higher estimates. For instance, Burnett et al. (2018) reported 8.9 (95% CI: 7.5–10.3) million premature deaths in 2015 while Huang et al. (2020) had estimated 9.3 (95% CI: 8.3–10.2) million premature deaths in 2010. In 2015 Liu et al. (2021) estimated 1.8 million premature deaths in China. It was also found that emissions fell in China post‐2013 due to strict policies (Figure 10), yet mortality rose 31% from 2005 to 2017 due to an aging population. Meanwhile, India, Africa, and Southeast Asia saw worsening pollution from urbanization, industrial growth, and biomass burning, while North America and Europe saw emission declines due to regulations. These trends explain why Lelieveld et al. (2015) estimated 3.3 million deaths in 2010. Liu et al. (2021) showed rising mortality despite lower emissions, and Burnett et al. (2018) estimated 8.9 million deaths in 2015, 30% higher than prior models.

The annual deaths of GBD 2019 for the year 2015 associated with NCD + LRI were 41.3 million, with our estimated ambient annual premature mortality attributable to PM_2.5_ and O_3_ exposure accounting for 13%, 14%, and 11%, for CESM, CAMS and ECLIPSE, respectively (GBD 2019 Risk Factors Collaborators, 2020; Xiong, Partha, et al., 2022). Similarly, the percentages of the annual YLL in 2015 from CESM, CAMS and ECLIPSE relative to the estimates of GBD 2019 in 2015 were 18%, 19% and 17%, respectively.

The disparities in estimates could stem from various factors, including differences in CTM, emissions inventories, and methodologies for assessing health burdens. Given that studies on human health burden assessments employ either the integrated exposure‐response (IER) or GEMM, our utilization of GEMM may shed light on disparities from prior research as the GEMM method effectively addressed the limitations of the IER approach (Burnett et al., 2018). A limitation for GEMM and IER is the assumption of uniform toxicity across all PM_2.5_ compositions. These models estimate mortality risk based on total mass concentration‐without differentiating between chemical composition or source specific toxicity. This may obscure the regional differences in health impacts driven by variations in PM_2.5_ composition (Burnett et al., 2018; Dominici et al., 2019; GBD 2019 Risk Factors Collaborators, 2020; Maji, 2020; Stanek et al., 2011). Assuming uniform toxicity may misrepresent health risks by ignoring differences in PM_2.5_ compositions.

Maji (2020) found that premature mortality estimates using the GEMM models for China surpassed those that used IER models by 104% and 61%, respectively. Pope et al. (2018) highlighted that in heavily polluted regions, the IER might underestimate the health impacts linked to PM_2.5_. This is relevant for regions such as India and China.

For example, Liu et al. (2021) utilized GEMM and estimated APD due to ambient PM_2.5_ exposure for China was 1.4 million in 2005 and 1.8 million in 2017. In contrast, studies using IER reported 1.0 (Zhao et al., 2017) and 1.3 million APDs (Wang et al., 2018) for 2010. Comparing these to our estimates of 1.2 (95% CI: 1.1–1.4), 1.5 (95% CI: 1.4–1.7) and 1.1 (95% CI: 1.0–1.2) million APD attributable to ambient PM_2.5_ for CEDS, CAMS and ECLIPSE, respectively, we observe that the IER models yield lower estimates.

A study by J. Liu et al. (2020), using Multi‐resolution Emission Inventory for China (MEIC) and MIX emission inventory (Li et al., 2017) along with the GEMM and Weather Research and Forecasting model and the Community Multiscale Air Quality Modeling System, estimated APDs attributed to anthropogenic PM_2.5_ as 1.6 (95% CI: 1.1–2.0) million in 2015. This estimate is significantly higher than the estimates in our study for China region: 0.6 million (95% CI: 0.5–0.7) from CEDS, 0.9 million (95% CI: 0.8–1.0) from CAMS, and 0.5 million (95% CI: 0.4–0.6) from ECLIPSE.

Regionally, the findings in our study indicate that India, China, and the ROA, experienced the highest proportion of premature deaths associated with PM_2.5_ + O_3_ exposure, at 33.8% (95% CI: 33.7%–33.9%), 38.9% (95% CI: 33.7%–47.1%) and 30.2% (95% CI: 30.0%–30.4%) of premature deaths for CEDS, CAMS and ECLIPSE respectively. We found that majority of PM_2.5_‐ and O_3_‐induced premature deaths occur in Asia and Africa. However, due to limited ground observational data in these regions, there may be over or underestimations in air quality impacts and mortality calculations (Salameh et al., 2016; Shami et al., 2022).

Future human health impact studies in the SSA and NAME regions are crucial, given our estimates of total global PM_2.5_ + O_3_ premature deaths at 5.5% (95% CI: 5.1–5.7), 5.0% (95% CI: 4.2–5.8), 6.0 (95% CI: 5.3–7.8) for CEDS, CAMS and ECLIPSE. In 2015, the NAME super‐region exhibited the highest death rates due to air pollution (Abbasi‐Kangevari et al., 2023). Similarly in 2019 SSA region estimated 16.3% deaths due to air pollution (Fisher et al., 2021a, 2021b).

Huang et al. (2021) had highlighted that large share of residential energy in low and middle‐income countries, all categorized as “Developing” in this study, relies on solid biofuel for cooking, heating, and lighting. WHO estimates that 90% deaths due to outdoor air pollution occur mainly in Asia and Africa. It is estimated that 55.3% of the world's population are exposed to increased levels of PM_2.5_ (Shaddick et al., 2020) and this is expected to worsen due to globalization.

For example, India's major cities, located within the Indo‐Gangetic Plain region, experience multiday air pollution events annually due to high population and pollution levels, significantly contributing to PM_2.5_‐related premature deaths (Asher Ghertner, 2021; David et al., 2019; Roozitalab et al., 2021). Similarly, in China, the regions most impacted by PM_2.5_ pollution include the North China Plain and the Yangtze River Delta (Zhai et al., 2019; Zhang et al., 2022), where rapid urbanization and industrial activities lead to severe air quality issues, resulting in high rates of premature deaths related to PM_2.5_ exposure.

**Figure 11:**
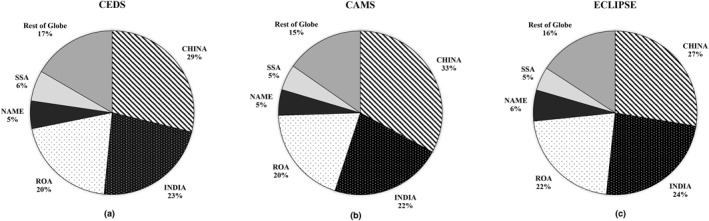
Pie chart of percentages of PM_2.5_ + O_3_ annual total premature deaths for Rest of Globe (LATIN, ECEUROPE, WEUROPE, USA, CANADA and Rest of the world), China, NAME, Rest of Asia, India and SSA for (a) Community Emissions Data System, (b) Copernicus Atmosphere Monitoring Service and (c) Evaluating of the Climate and Air Quality Impacts of Short‐Lived Pollutants.

This burden disproportionately affects the most vulnerable populations, including low‐income individuals and those in developing nations (Galimova et al., 2022; Gardner‐Frolick et al., 2022). Our study shows that 79.5% (CEDS), 81.2% (CAMS), and 77.6% (ECLIPSE) of premature deaths due to anthropogenic activities are associated with Asia and Africa continent. Figure 11 shows an illustration of percentage for Asia and Africa in CEDS, CAMS and ECLIPSE. In the context of Asian and African nations (China, India, ROA, NAME and SSA) exclusively, this study reveals a consistent pattern across the three inventories.

Lastly, we acknowledge the discrepancies with previous studies due to different methodologies and emission inventories, as previously mentioned. Despite this, our study shows a notable trend: estimated global PM_2.5_ mortality is three times higher than the O_3_ mortality, indicating that PM_2.5_ is the primary contributor of the outdoor air pollution‐related global health burden. This aligns with a previous study by Anenberg et al. (2010), which reported that their estimated PM_2.5_ mortality was five times higher than the O_3_ mortality.

We also recognize that, in addition to the differences in emission inventories explored here, surface PM_2.5_ + O_3_ and premature deaths attributed to PM_2.5_ + O_3_, are model‐dependent and influenced by the GBD study utilized. If available, it is recommended to use localized emission inventories, recognizing that no inventory is entirely precise (Madrazo et al., 2018), yet such an approach could enhance emission analysis. The limitations in data and methodologies utilized for developing these inventories, as highlighted by Miller et al. (2006) can significantly impact air quality management.

## 4 Conclusions

We investigated how differences in emissions inventories (CEDS, CAMS, and ECLIPSE) affect air quality and human health impact estimates in 2015 using CAM‐chem. This study is the first study contrasting these three emission inventories and quantifying the global human health impacts attributable to PM_2.5_ and O_3_ exposure due to anthropogenic activities.

Due to existing uncertainties in anthropogenic emission inventories, this significantly impacts the accuracy of health impact assessments. As evidenced by our intercomparisons using CEDS as the baseline, we found substantial discrepancies in health impact estimates when using different emission inventories. For example, we found that ECLIPSE APDs and air quality impacts were significantly lower. We had found that PM_2.5_‐ and O_3_‐induced premature deaths for the year 2015 to be 2.5 (95% CI: 2.1–3.1), 2.8 (95% CI: 2.4–3.4), and 1.9 (95% CI: 1.6–2.4) million for CEDS, CAMS and ECLIPSE respectively. The regions with the highest APDs are China, India, and ROA. The corresponding YLL associated with PM_2.5_ + O_3_ in 2015 were calculated to be 55.4 (95% CI: 50.6–64.1), 62.7 (95% CI: 54.5–70.6) and 62.1 (95% CI: 45.2–85.4) million years for CEDS, CAMS, and ECLIPSE, respectively. This illustrates how differences in emission data can lead to uncertainties in estimating health impacts. Upon validating the model, we found a much larger negative NMB for Europe, India, and China using the ECLIPSE emissions inventory, indicating a significant underprediction by the model. We found that discrepancies in emissions inventories contributed to differences in human health impact estimates, highlighting the critical need for accurate emission inventories to improve health impact assessments and inform effective air quality policies. This issue is pronounced in China and India, two regions with large populations, where existing underestimations contribute significantly to bias in our estimates of the associated health burden.

Our findings highlight the differences when using commonly used emission inventories to investigate air quality and human health impacts. Additionally, we identified significant disparities in the impact of air pollution on premature deaths due to PM_2.5_ and O_3_ exposure. Developing nations experiencing an average impact that it is on average 11 times higher than that in Developed nations using CEDS, CAMS and ECLIPSE inventories. Our study not only offers valuable scientific evidence for global policymakers in their efforts to mitigate the impact of air quality on human health, but also highlights the importance of improving emissions inventories to ensure more accurate health impact assessments.

Given the existing discrepancies across inventories, an multi‐model ensemble approach may help reduce uncertainties by using multiple models with different assumptions, and chemical mechanisms (Fiore et al., 2009; Pachón et al., 2024; Thornhill et al., 2021; Van Donkelaar et al., 2016). Using a multi‐model ensemble may help mitigate individual model biases. By averaging across simulations, it has the potential to align more closely with ground‐based observations and GBD 2019 mortality estimates, thus offering a more robust assessment of population exposure and the associated health burden than relying on a single emission inventory (Deroubaix et al., 2024; Pozzer et al., 2023). Improving emissions estimates in regions with limited observational data remains a key priority and future efforts would benefit from integrating ground‐based observations with satellite observations (Miyazaki et al., 2020). Furthermore, it is important to evaluate how different inventories perform across regions, as their accuracy depends on data availability and emission sources.

Lastly, interdisciplinary collaboration between atmospheric scientists, public health experts, and policymakers is crucial for translating air quality modeling results into actionable public health interventions (Abera et al., 2024; Fisher et al., 2021a, 2021b; Holloway et al., 2024). Future studies should also consider exploring approaches that account for regional health vulnerabilities, particularly in the Global South, where pollution burdens are disproportionately high. Addressing these gaps is critical to implementing effective air quality regulations and reducing premature deaths globally.

## Conflict of Interest

The authors declare no conflicts of interest relevant to this study.

## Supporting information

Supporting Information S1

## Data Availability

Data of CESM CAM6‐Chem model‐simulated PM_2.5_ and O_3_ concentrations and associated premature deaths in 2015 are available publicly at Salah (2025).
