# Supplementary material for: Impact of Anthropogenic Emission Estimates on Air Quality and Human Health Effects
Source: Geohealth. 2025 Oct 21;9(10):e2024GH001223. doi: 10.1029/2024GH001223 (PMC12538235; doi:10.1029/2024GH001223)
Supplement: Supplementary file 1 — Supporting Information S1 [file GH2-9-e2024GH001223-s001.pdf]

Supplementary Material for

Impact of anthropogenic emission estimates on air quality and human health effects

Halima Salah[1], Ying Xiong[1,2], Debatosh Partha[1,3], Noribeth Mariscal[1,4], Like Wang[1], Simone Tilmes[5], Wenfu Tang[5], and Yaoxian Huang[1,*]

[1]Department of Civil and Environmental Engineering, Wayne State University, Detroit, MI, USA

[2]Now at College of Environment and Climate, Institute for Environmental and Climate Research, Guangdong-Hongkong-Macau Joint Laboratory of Collaborative Innovation for Environmental Quality, Jinan University, Guangzhou, China

[3]Now at Department of Earth, Environmental and Planetary Sciences, Northwestern University, Evanston, IL, USA

[4]Now at Max-Planck-Institute for Meteorology, Hamburg, Germany

[5]NSF National Center for Atmospheric Research, Boulder, CO, USA

[*]*Correspondence to:* Yaoxian Huang (yaoxian.huang@wayne.edu)

Number of Pages (including this cover page): 17
Number of Texts: 3
Number of Figures: 8
Number of Tables: 7

Text S1. Emissions Inventories and CAM6-Chem Model Description
Emission Inventories

The Earth System is the dynamic interaction of physical, chemical, and biological processes (Flato, 2011). Since we are unable to perform tests on the actual Earth System due to natural constraints, the alternative is to create practical computer models that enable us to research the system's dynamic relationships and behavior, as well as its responses to contaminants. To understand the relationship between pollution and various sources, atmospheric chemistry transport models are used (McDuffie et al., 2020). In global analysis and simulation, several global pollution inventories have been used. A bottom-up approach is used to build all three inventories. For each emitted compound, are measured using observed activity data (e.g., energy consumption) and source- and region-specific emission factors. The most used inventories are the three commonly used global emissions inventories; Community Emissions Data System (CEDS) (Hoesly et al., 2018; McDuffie et al., 2020), Copernicus Atmosphere Monitoring Service (CAMS) (Soulie et al., 2022) and Evaluating the CLimate and Air Quality ImPacts of ShortlivEd Pollutants (ECLIPSE) (Amann et al., 2011). A bottom-up approach is used to build all three inventories. For each emitted compound, are measured using observed activity data (e.g., energy consumption) and source- and region-specific emission factors. ECLIPSE from the GAINS (Greenhouse gases Air pollution Interactions and Synergies) model that has been developed by the International Institute for Applied Systems Analysis (IIASA) (https://iiasa.ac.at). Global emissions (for both historical and near-future times) are available in 5 five-year increments and include 160 country regions (Stohl et al., 2015). For the Coupled Model Intercomparison Project phase 5 (CMIP5), Lamarque et al. (2010) created a historical data set that includes global, gridded estimates of anthropogenic and open burning emissions at 10-year intervals from 1850 to 2000. In our study the CEDS data we have been using is consists of sectoral and gridded historical inventory of aerosols, GHGs and reactive gases-not including agricultural waste burning- for use in Coupled Model Intercomparison Project phase 6 (CMIP6).

Table S1. CEDS working sectors adapted from Hoesly et al. (2018) and Xiong et al. (2022).

CEDS working sectors		
**Energy production**	1A2g_Ind-Comb-other	**Residential, Commercial, Other**
1A1a_Electricity-public	2A1_Cement-production	1A4a_Commercial-institutional
1A1a_Electricity-autoproducer	2A2_Lime-production	1A4b_Residential
1A1a_Heat-production	2Ax_Other-minerals	1A4c_Agriculture-forestry-fishing
1A1bc_Other-transformation	2B_Chemical-industry	1A5_Other-unspecified
1B1_Fugitive-solid-fuels	2C_Metal-production	**Agriculture**
1B2_Fugitive-petr-and-gas	2-D_Other-product-use	3B_Manure-management
1B2d_Fugitive-other-energy	2-D_Paint-application	3-D_Soil-emissions
7A_Fossil-fuel-fires	**Transportation**	3I_Agriculture-other
**Industry**	1A3ai_International-aviation	3-D_Rice-Cultivation
1A2a_Ind-Comb-Iron-steel	1A3aii_Domestic-aviation	3E_Enteric-fermentation
1A2b_Ind-Comb-Non-ferrous-metals	1A3b_Road	**Shipping**
1A2c_Ind-Comb-Chemicals	1A3c_Rail	1A3di_International shipping
1A2d_Ind-Comb-Pulp-paper	1A3di_International-shipping	1A3di_Oil_tanker_loading
1A2e_Ind-Comb-Food-tobacco	1A3di_Oil_tanker_loading	**Waste**
1A2f_Ind-Comb-Non-metalic-minerals	1A3dii_Domestic-navigation	5A_Solid-waste-disposal
1A2g_Ind-Comb-Construction	1A3eii_Other-transp	5E_Other-waste-handling
1A2g_Ind-Comb-transpequip		5C_Waste-combustion
1A2g_Ind-Comb-machinery		5-D_Wastewater-handling
1A2g_Ind-Comb-mining-quarying		6A_Other-in-total
1A2g_Ind-Comb-wood-products		6B_Other-not-in-total
1A2g_Ind-Comb-textile-leather		

Table S2. CEDS emission inventory sector emissions for the year 2015 (kt/yr).

Sectors \ Species	NO_x	BC	CO	NMVOC	NH_3	SO_2	OC
**AGR**	5082.1	0.0	0.0	7384.6	44933.7	0	0
**ENE**	29308.2	601.0	61704.7	34444.7	1338.6	43067.4	999.0
**IND**	18432.7	898.4	91286.7	11189.2	1087.7	30255.5	1677.3
**TRA**	39027.4	1327.8	159318.0	30665.1	366.8	3030.2	552.0
**SLV**	0	0	0	30092.2	0	0	0
**WST**	2474.2	272.6	17400.5	2780.4	8452.6	240.2	1713.1
**SHP**	18875.6	75.0	729.4	3099.7	17.7	9741.7	57.0
**RCO**	8665.9	2866.8	235119.0	29916.4	3213.4	5724.4	8857.8
**Total**	121866.1	6041.6	565558.3	149572.2	59410.7	92059.5	13856.3

Table S3. CAMS emissions inventory and calculation for NO_2.

CAMS	Total Emissions (kt/yr)
NO_2	121473
BC	4772
CO	578126
NMVOC	148307
NH_3	49097
SO_2	99724
OC	16541

Convert to NOx (NO_2) = NO*46/30

NO_2 = 79222*46/30 =121473

Table S4. ECLIPSE calculation: International Shipping and Updated Shipping.

ECLIPSE Emissions (kt/yr)	NO_x	BC	CO	VOC	NH_3	SO_2	OC
Total Emissions	124895.2	6351.5	548381.3	112031.0	60986.0	73335.1	13763.2
International Shipping	19082.3	42.5	3057.3	605.9	0.0	10150.8	280.6
Updated Shipping	19177.9	90.4	3072.8	609.0	0.0	10196.6	306.9
Net Emissions	124990.8	6399.4	548396.8	112034.0	60986.0	73380.9	13789.5

Note: Net Emissions= Total Emissions + (International Shipping-Updated Shipping)

CAM6-Chem Model Description

We ran the CAM6-Chem model simulations from January 1, 2014 to January 1, 2016, with the horizontal resolution of 0.95° latitude by 1.25° longitude. The 2015 modeling outputs are used for result analysis and the rest used for spin-up. CAM6-Chem is nudged with Modern-Era Retrospective analysis for Research and Applications version 2 (MERRA-2) meteorological data sets, with each model time step of 30 minutes. This contains 32 vertical layers from surface up to abut 40km.

Text S2. Air Quality Impacts: Annual Mean Concentrations

This section extends the discussion from section 3.2 and 3.3, where we utilized CESM2 CAM6-Chem to assess the influence of both human-made and natural emissions on the quality of the surrounding air and provides the referenced annual mean concentrations. To reiterate section 3.2, in this research, our primary focus is on utilizing the CEDS, CAMS and ECLIPSE as detailed anthropogenic emission inventories. Our analysis primarily centers on evaluating the global impact of various air quality pollutants, including NO_x, BC, $PM_{2.5}$, CO, NMVOC, NH_3 and SO_2 at the Earth's surface in 2015. When examining the difference in spatial distribution of global annual mean surface concentrations for $PM_{2.5}$ (Figure S6) and O_3 (Figure S7) for the year 2015, using CEDS as the baseline, we found that CAMS showed a more positive difference in East and South Asia, as well as in North America in North America and in South America. However, when compared to ECLIPSE, we found that the difference being negative in most regions.

Table S5. Regional annual mean concentrations of $PM_{2.5}$ for CEDS, CAMS and ECLIPSE. Unit: $\mu g/m^3$.

REGION	CEDS	CAMS	ECLIPSE
INDIA	5.5	8.0	3.9
CHINA	0.2	0.2	0.2
NAME	0.9	0.7	0.6
ROA	13.9	14.2	11.1
SSA	1.3	1.4	1.0
LATIN	3.2	2.9	2.1
WEUROPE	3.2	3.3	2.3
ECEUROPE	0.1	0.2	0.0
USA	1.9	2.0	2.0
CANADA	0.9	1.3	0.7
ROW	1.2	1.3	0.8

Text S3. Validations of Model Simulations Against Observations

Figure 2 found that the model simulated $PM_{2.5}$ concentrations, across the four regions (US, Europe, India, and China) for the three inventories, agreed with the observations within a factor of 2.0, 1.7 and 2.3 with an averaged NMB of -40.2%, -34.2% and -47.4% for CEDS, CAMS and ECLIPSE respectively. We have found that the modeled concentration for the US agreed with the observation data with NMB of -7.4% (CEDS), -1.6% (CAMS) and -13.5% (ECLIPSE). Meanwhile, the model's simulations of $PM_{2.5}$ levels in China were found to be less accurate; we found a much higher underestimation with NMB at -66.1% (CEDS), -53.5% (CAMS), and -75.3%. (ECLIPSE). NMB is calculated as $NMB = \left[\sum_i (M_i - O_i) \Big/ \sum_i (O_i) \right] \times 100\%$, where M and O are the modeled and observed values, respectively, and i represents observational site in each region.

Table S6. Percentage difference of total APD per region attributed to $PM_{2.5}$ using CEDS as baseline.

	$PM_{2.5}$	
**REGION**	**CAMS**	**ECLIPSE**
CHINA	41.5%	-23.9%
CANADA	9.9%	-4.5%
ECEUROPE	-3.9%	-15.9%
INDIA	1.9%	-13.6%
LATIN	-2.7%	-28.0%
NAME	-10.8%	-23.4%
ROA	3.3%	-22.9%
ROW	2.9%	-19.6%
SSA	12.4%	13.6%
USA	22.8%	-12.3%
WEUROPE	4.0%	-23.5%

Table S7. Percentage difference of total APD per region attributed to O_3 using CEDS as baseline.

	O_3	
**REGION**	**CAMS**	**ECLIPSE**
CHINA	-10.0%	-57.0%
CANADA	-5.1%	-27.5%
ECEUROPE	-2.0%	-25.4%
INDIA	4.4%	-32.1%
LATIN	-1.7%	-24.8%
NAME	0.0%	-18.5%
ROA	-6.9%	-33.7%
ROW	-24.6%	-48.1%
SSA	2.3%	-9.8%
USA	-1.9%	-24.7%
WEUROPE	-8.4%	-29.9%

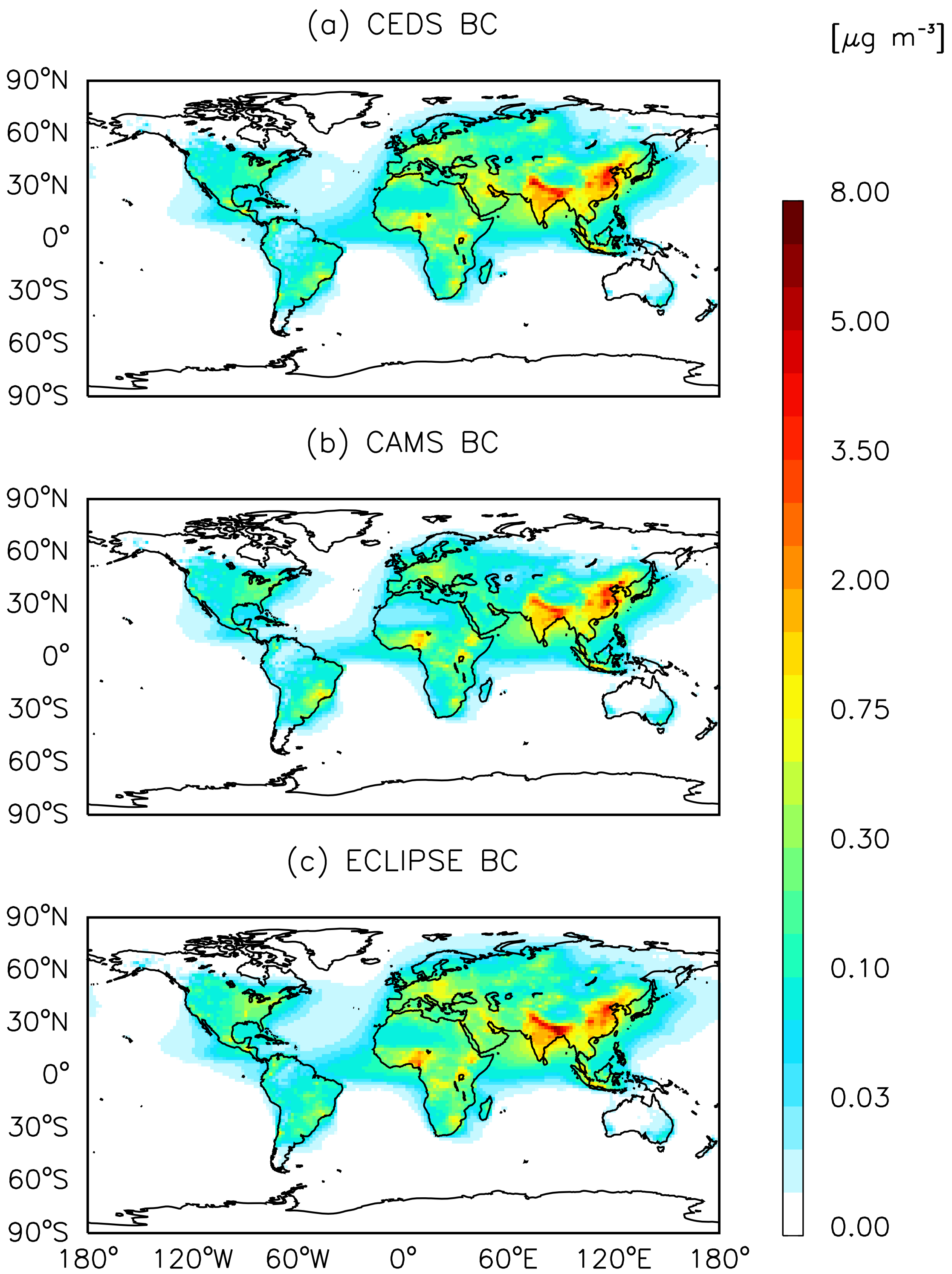

Figure S1. Global annual mean surface BC concentrations for the year 2015 from CESM2.2 CAM6-Chem using (a) CEDS, (b) CAMS and (c) ECLIPSE.

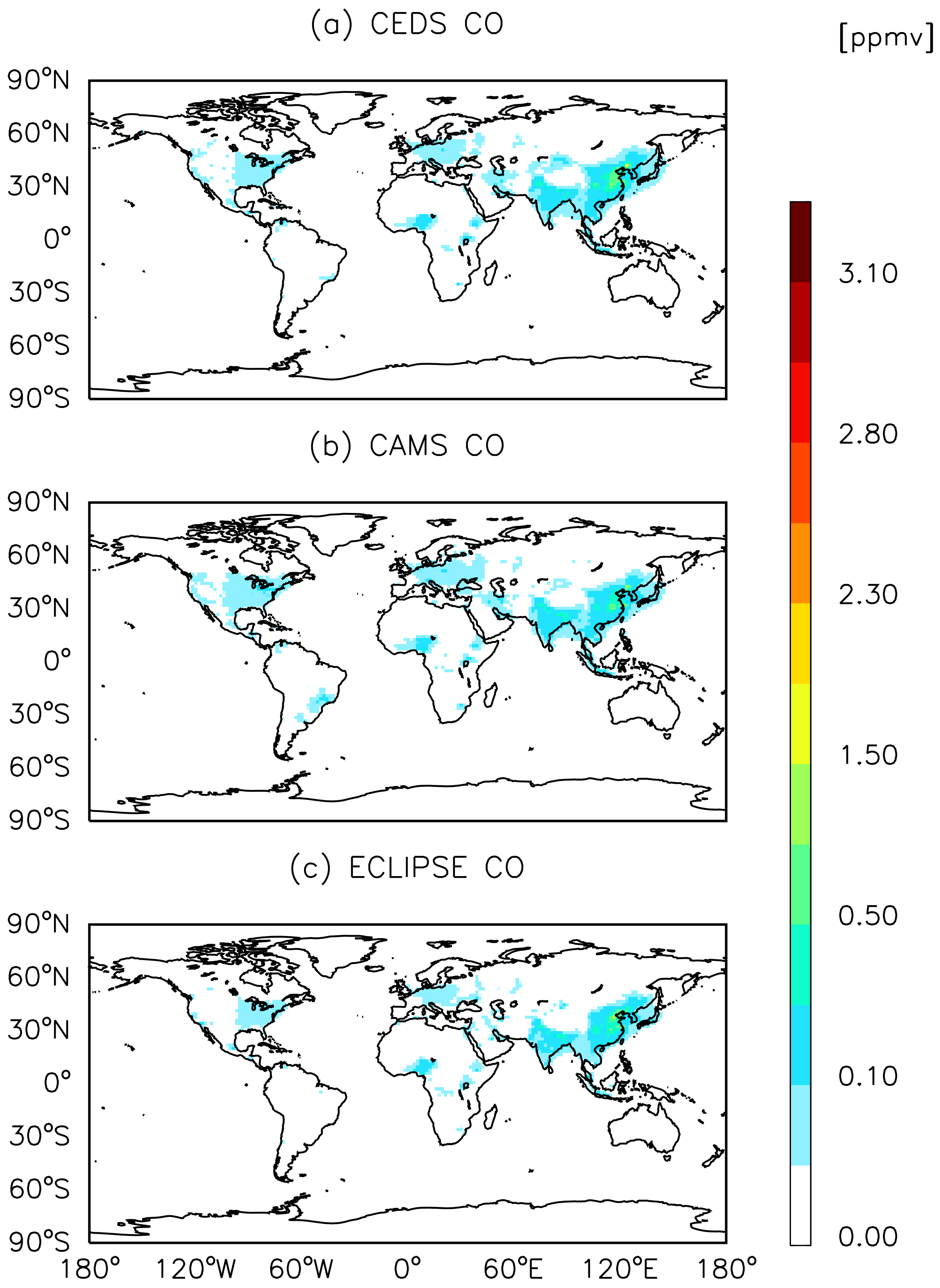

Figure S2. Global annual mean surface CO concentrations for the year 2015 from CESM2.2 CAM6-Chem using (a) CEDS, (b) CAMS and (c) ECLIPSE.

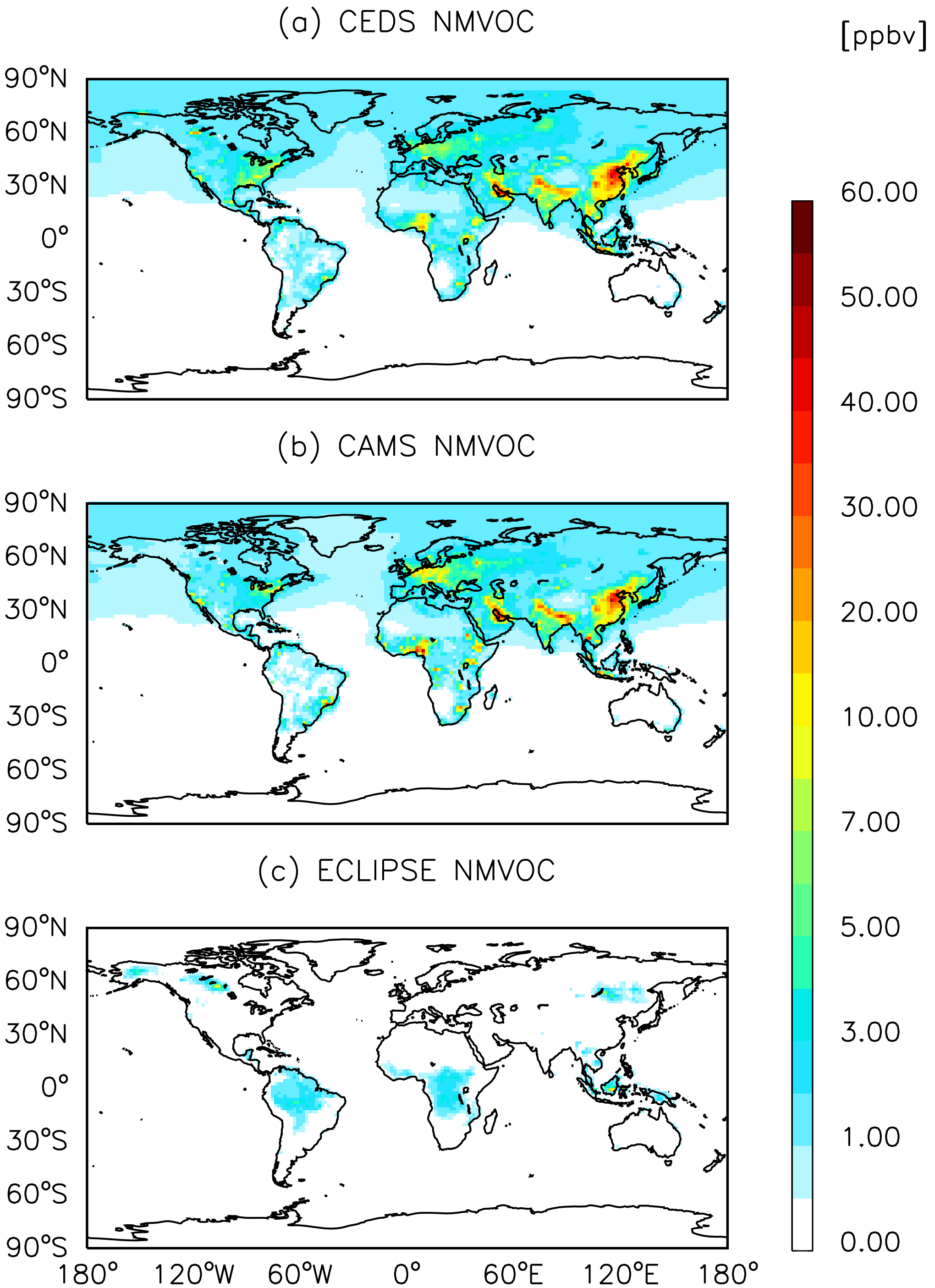

Figure S3. Global annual mean surface NMVOC concentrations for the year 2015 from CESM2.2 CAM6-Chem using (a) CEDS, (b) CAMS and (c) ECLIPSE.

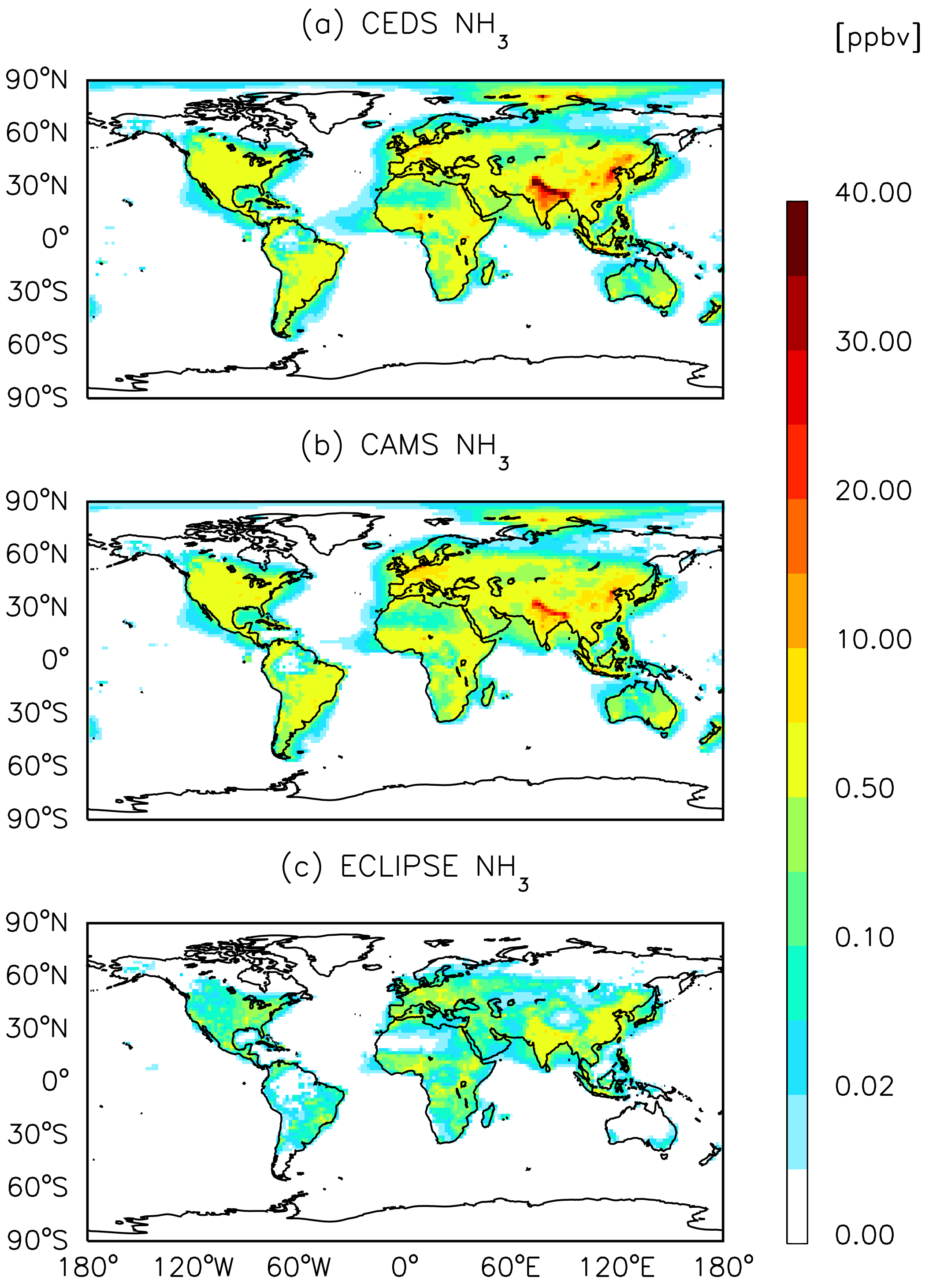

Figure S4. Global annual mean surface NH_3 concentrations for the year 2015 from CESM2.2 CAM6-Chem using (a) CEDS, (b) CAMS and (c) ECLIPSE.

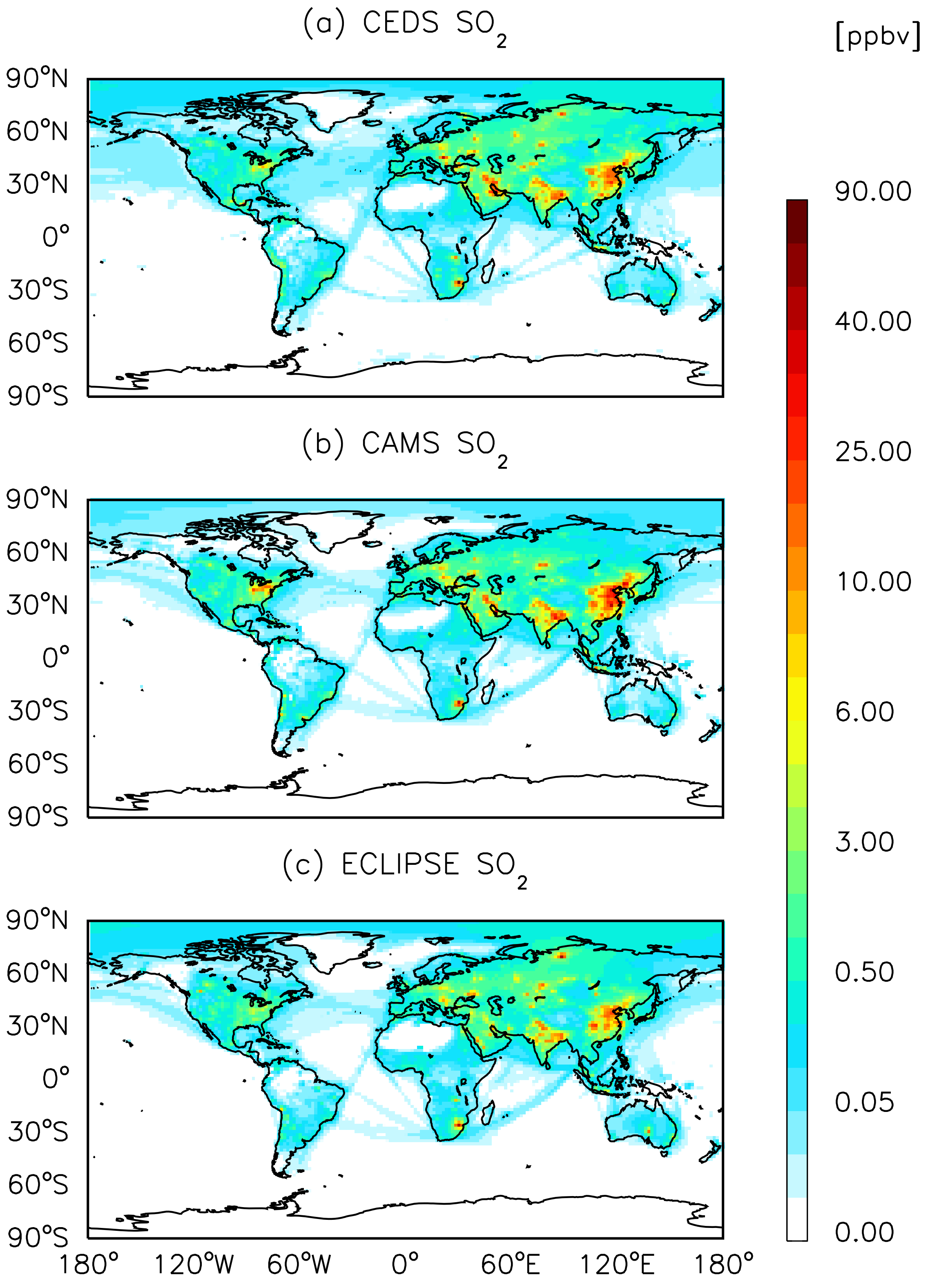

Figure S5. Global annual mean surface SO_2 concentrations for the year 2015 from CESM2.2 CAM6-Chem using (a) CEDS, (b) CAMS and (c) ECLIPSE.

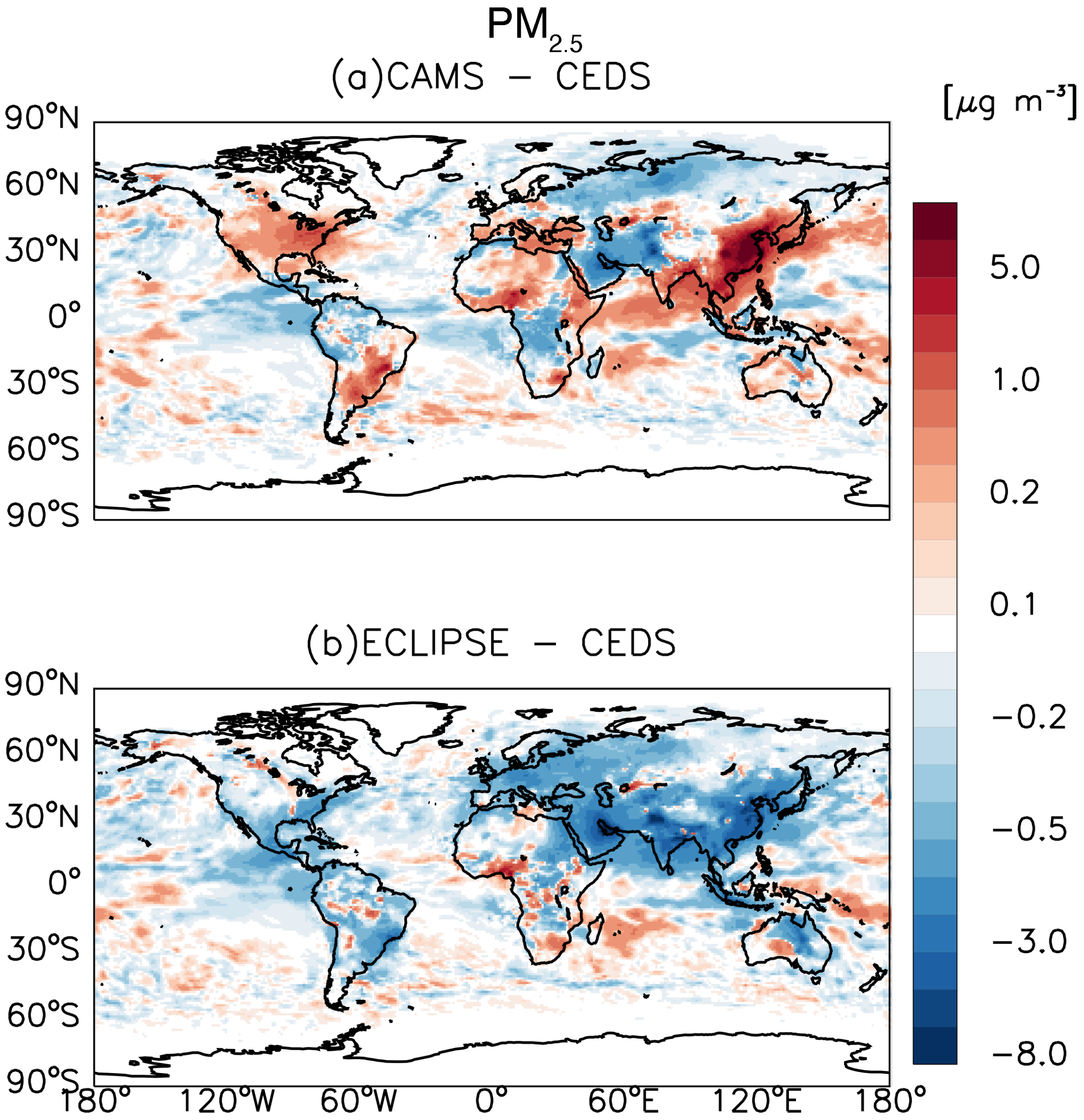

Figure S6. Differences of spatial distribution of global annual mean surface $PM_{2.5}$ concentrations for the year 2015 (a) between CAMS and CEDS (CAMS-CEDS), and (b) between ECLIPSE and CEDS (ECLIPSE-CEDS), with CEDS as baseline. Units: μg/m^3.

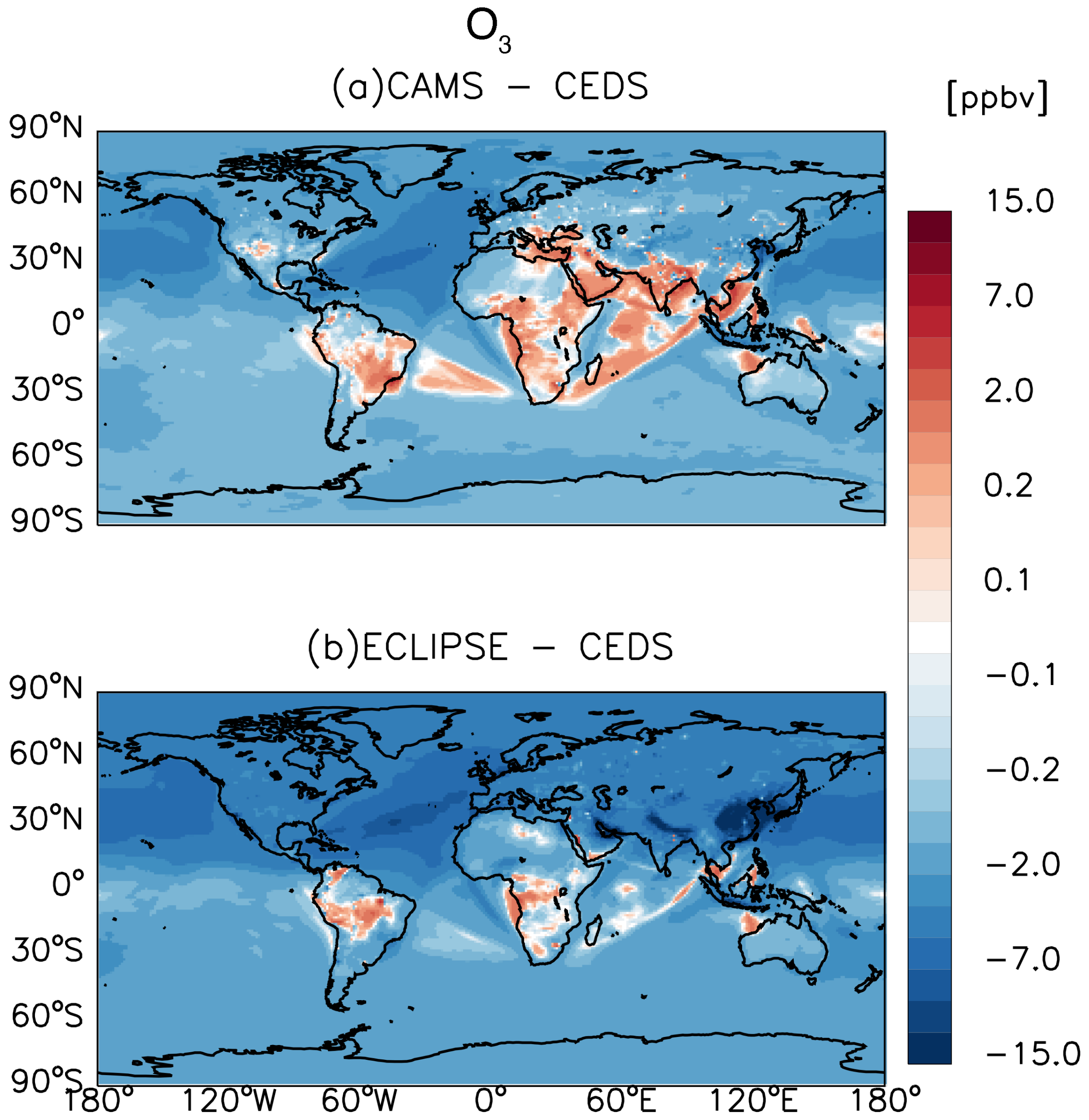

Figure S7. Differences of spatial distribution of global annual mean surface O_3 concentrations for the year 2015 (a) between CAMS and CEDS (CAMS-CEDS), and (b) between ECLIPSE and CEDS (ECLIPSE-CEDS), with CEDS as baseline. Units: ppbv.

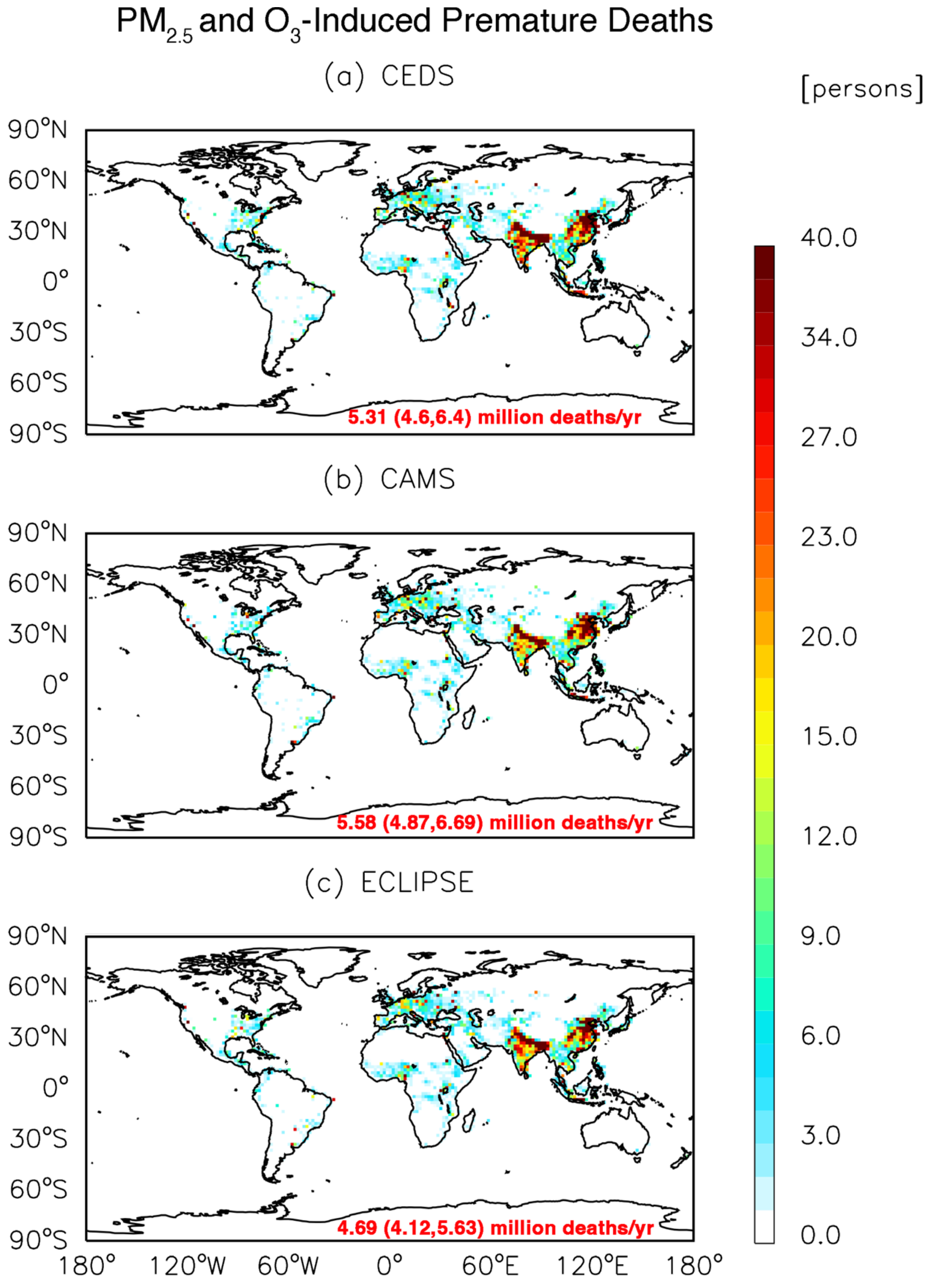

Figure S8. Global annual total $PM_{2.5}$- and O_3-induced premature deaths due to ambient air pollution for the year 2015, for (a) CEDS, (b) CAMS and (c) ECLIPSE. Parenthesis shows the uncertainty and 95% confidence intervals

References

Amann, M., Bertok, I., Borken-Kleefeld, J., Cofala, J., Heyes, C., Höglund-Isaksson, L., Klimont, Z., Nguyen, B., Posch, M., Rafaj, P., Sandler, R., Schöpp, W., Wagner, F., & Winiwarter, W. (2011). Cost-effective control of air quality and greenhouse gases in Europe: Modeling and policy applications. *Environmental Modelling and Software*, *26*(12), 1489–1501. https://doi.org/10.1016/j.envsoft.2011.07.012

Flato, G. M. (2011). Earth system models: An overview. *Wiley Interdisciplinary Reviews: Climate Change*, *2*(6), 783–800. https://doi.org/10.1002/wcc.148

Hoesly, R. M., Smith, S. J., Feng, L., Klimont, Z., Janssens-Maenhout, G., Pitkanen, T., Seibert, J. J., Vu, L., Andres, R. J., Bolt, R. M., Bond, T. C., Dawidowski, L., Kholod, N., Kurokawa, J. I., Li, M., Liu, L., Lu, Z., Moura, M. C. P., O'Rourke, P. R., & Zhang, Q. (2018). Historical (1750-2014) anthropogenic emissions of reactive gases and aerosols from the Community Emissions Data System (CEDS). *Geoscientific Model Development*, *11*(1), 369–408. https://doi.org/10.5194/gmd-11-369-2018

Huang, Y., Partha, D. B., Harper, K., & Heyes, C. (2021). Impacts of Global Solid Biofuel Stove Emissions on Ambient Air Quality and Human Health. *GeoHealth*, *5*(3), 1–16. https://doi.org/10.1029/2020GH000362

Lamarque, J. F., Bond, T. C., Eyring, V., Granier, C., Heil, A., Klimont, Z., Lee, D., Liousse, C., Mieville, A., Owen, B., Schultz, M. G., Shindell, D., Smith, S. J., Stehfest, E., Van Aardenne, J., Cooper, O. R., Kainuma, M., Mahowald, N., McConnell, J. R., … Van Vuuren, D. P. (2010). Historical (1850-2000) gridded anthropogenic and biomass burning emissions of reactive gases and aerosols: Methodology and application. *Atmospheric Chemistry and Physics*, *10*(15), 7017–7039. https://doi.org/10.5194/acp-10-7017-2010

Lamarque, J. F., Emmons, L. K., Hess, P. G., Kinnison, D. E., Tilmes, S., Vitt, F., Heald, C. L., Holland, E. A., Lauritzen, P. H., Neu, J., Orlando, J. J., Rasch, P. J., & Tyndall, G. K. (2012). CAM-chem: Description and evaluation of interactive atmospheric chemistry in the Community Earth System Model. *Geoscientific Model Development*, *5*(2), 369–411. https://doi.org/10.5194/gmd-5-369-2012

McDuffie, E. E., Smith, S. J., O'Rourke, P., Tibrewal, K., Venkataraman, C., Marais, E. A., Zheng, B., Crippa, M., Brauer, M., & Martin, R. V. (2020). A global anthropogenic emission inventory of atmospheric pollutants from sector- And fuel-specific sources (1970-2017): An application of the Community Emissions Data System (CEDS). *Earth System Science Data*, *12*(4), 3413–3442. https://doi.org/10.5194/essd-12-3413-2020

Soulie, A., Granier, C., Gon, H. D. V. A. N. D. E. R., Kuenen, J. J. P., Darras, S., Doumbia, T., Galle, B., Guauss, M., Guevara, M., Jalkanen, J., Keita, S., Liousse, C., Doubalova, J., Simpson, D., & Sindelarova, K. (2022). *THE COPERNICUS ATMOSPHERE MONITORING SERVICE (CAMS) EMISSIONS OF GREENHOUSE GASES AND AIR POLLUTANTS Global anthropogenic emissions : CAMS-GLOB-ANT*.

Stohl, A., Aamaas, B., Amann, M., Baker, L. H., Bellouin, N., Berntsen, T. K., Boucher, O., Cherian, R., Collins, W., Daskalakis, N., Dusinska, M., Eckhardt, S., Fuglestvedt, J. S., Harju, M., Heyes, C., Hodnebrog, Hao, J., Im, U., Kanakidou, M., … Zhu, T. (2015). Evaluating the climate and air quality impacts of short-lived pollutants. *Atmospheric Chemistry and Physics*, *15*(18), 10529–10566. https://doi.org/10.5194/acp-15-10529-2015

Tilmes, S., Lamarque, J. F., Emmons, L. K., Kinnison, D. E., Ma, P. L., Liu, X., Ghan, S., Bardeen, C., Arnold, S., Deeter, M., Vitt, F., Ryerson, T., Elkins, J. W., Moore, F., Spackman, J. R., & Val Martin, M. (2015). Description and evaluation of tropospheric chemistry and aerosols in the Community Earth System Model (CESM1.2). *Geoscientific Model Development*, *8*(5), 1395–1426. https://doi.org/10.5194/gmd-8-1395-2015
